# Prevalence of Common Mental Disorders in South Asia: A Systematic Review and Meta-Regression Analysis

**DOI:** 10.3389/fpsyt.2020.573150

**Published:** 2020-09-02

**Authors:** Sadiq Naveed, Ahmed Waqas, Amna Mohyud Din Chaudhary, Sham Kumar, Noureen Abbas, Rizwan Amin, Nida Jamil, Sidra Saleem

**Affiliations:** ^1^ Department of Child Psychiatry, Kansas University Medical Center, Kansas City, KS, United States; ^2^ Institute of Population Health, University of Liverpool, Liverpool, United Kingdom; ^3^ Nishtar Medical University, Multan, Pakistan; ^4^ Dow Medical College, Karachi, Pakistan; ^5^ FMH College of Medicine and Dentistry, Lahore, Pakistan; ^6^ King Edward Medical University, Lahore, Pakistan; ^7^ Fatima Jinnah Medical University, Lahore, Pakistan

**Keywords:** psychiatric illness, South Asia, prevalence, epidemiology, common mental disorders

## Abstract

South Asian countries report the highest prevalence of common mental disorders (CMDs) globally. This systematic review and meta-analysis report the pooled prevalence of CMDs among the South Asian countries. Database searches were conducted in eight electronic databases. Titles, abstracts, full-text screening, and extraction of data on the event rate of 17 indicators of CMDs were performed by two independent reviewers. A total of 160 studies were included and data analysis was done using the Comprehensive Meta-analysis Software (v.3). A prevalence of depressive symptoms was 26.4% among 173,449 participants, alcohol abuse was 12.9% (n = 107,893); anxiety 25.8% (n = 70,058); tobacco smoking 18.6% (n = 84,965); PTSD 17.2% (n = 42,298); mixed anxiety and depression 28.4% (n = 11,102); suicidal behaviors 6.4% (n = 25,043); misuse of opiates 0.8% (n = 37,304); tobacco chewing 21.0% (n = 10,586); use of cannabis 3.4% (n = 10,977); GAD 2.9% (n = 70,058); bipolar disorder 0.6% (n = 7,197); IV drug abuse 2.5% (n = 15,049); panic disorder 0.01% (n = 28,087); stimulant use 0.9% (n = 1,414); OCD 1.6% (n = 8,784) and phobic disorders 1.8% (n = 27,754). This study reported a high prevalence of CMDs in South Asian countries; necessitating further research on psychiatric epidemiology in those contexts. It informs the need for effective policymaking and implementation of culturally appropriate multilevel interventions.

## Introduction

Mental disorders are highly prevalent across the globe, especially in low- and middle-income countries (LMICs) (1). The World Health Organization’s Mental Health Gap Action Programme (mhGAP) identifies depression, bipolar affective disorder, schizophrenia, and other psychotic disorders, dementia, intellectual disabilities, and developmental disorders like autism as priority mental and neurological disorders (1). These common mental disorders (CMDs) are associated with significant functional impairment as well as social and economic consequences (1). It is estimated that depression and anxiety disorders alone cost around one trillion dollars/year to the global economy (2). Although CMDs are common worldwide, a higher proportion (~80%) of people with mental health disorders reside in LMICs (3), where these account for 8.8% to 16.6% of the total burden of diseases (4). The negative implications of CMDs are further compounded by a high treatment gap; 76% to 85% of people with a mental disorder in low-income countries receive no treatment for their disorder compared to 35%, and 50% of people with mental disorders in high-income countries (1).

South Asian countries comprise one-quarter of the world’s population and include countries like India, Pakistan, Nepal, Sri Lanka, Bhutan, Bangladesh, Afghanistan, and the Maldives, comprise one-quarter of the world’s population (5, 6). Marred by high poverty rates approximately 150–200 million people in this region have a diagnosed psychiatric disorder and limited access to mental health (6). Despite the significant burden of illness, the mental health infrastructure in this region is relatively weak, with less than 1% of the total national budgets allocated to it (7). There is also a shortage of psychiatrists and other mental health professionals, clinical psychologists, as well as social workers (6). A study estimates that the median number of mental health providers in this region is 5.3 per 100,000 population, almost half of the overall global median (8). There is only one psychiatrist per 100,000 population in nine out of the 11 countries in this region (8). In addition to the limitations in resource allocation, the lack of community awareness toward mental health and prevailing stigma further limits the number of patients that actively seek health care (9).

South Asia has a diverse and rapidly growing population of people from different cultures, religions, and socioeconomic backgrounds (10). However, the information available regarding the prevalence of psychiatric disorders in South Asia is scant. In the past, a few studies have estimated the prevalence and burden of psychiatric disorders in this region. A meta-analysis reported that the total prevalence of mental disorders in India is 58.2 per 1,000 people (11). In Bangladesh, the prevalence of mental disorders varied from 6.5% to 31.0% among adults (12). The epidemiological data related to mental disorders for Sri Lanka, Afghanistan, and Bhutan is limited; only a few published articles provide an estimate of the prevalence of psychiatric illness in these countries. There is a lack of synthesized evidence on the epidemiological burden of CMDs in this region. This systematic review and meta-analysis acknowledged this knowledge gap and aimed to understand the prevailing trends of mental disorders in South Asian countries. The present review has sought to identify the literature on the prevalence of psychiatric disorders in the South Asian countries published over a decade. This information would be helpful for future policymaking and implementation of effective health care measures to improve mental health in the region.

## Materials and Methods

This systematic review was conducted as per the updated PRISMA guidelines (13) and the protocol registered in PROSPERO (CRD42019130662). An electronic search was conducted in eight electronic databases including PubMed, Scopus, ISI Web of Science, POPLINE, New York Academy of Medicine Grey Literature Report (NYAM), PsycINFO, PsycARTICLES and the Cumulative Index to Nursing and Allied Health Literature (CINAHL) on March 31, 2019, using following search terms:

[(“Prevalence” OR “Frequency” OR epidemiology OR epidemiological OR proportion) AND (“Mood disorder” OR “Depression” OR “Substance abuse” OR “substance use” OR “Posttraumatic stress disorder” OR PTSD OR “obsessive-compulsive disorder” OR “OCD” OR”bipolar disorder” OR “Anxiety” OR “Panic disorder” OR “schizophrenia” OR “GAD” OR “Acute stress disorders”) AND (“South Asia” OR “Afghanistan” OR “Nepal” OR “Pakistan” OR “India” OR “Sri Lanka” OR “Bhutan” OR “Bangladesh” OR “Maldives”)].

We only included studies that were published in the last 10 years, i.e., from 2009 to 2019. No restriction of language was applied. Three independent reviewers screened the databases for eligible studies based on their titles and abstracts, followed by the screening of full texts. All discrepancies among reviewers were resolved through discussion between reviewers and guidance from a senior author (SN).

### Inclusion and Exclusion Criteria

All studies reporting the prevalence of common psychiatric disorders among adults (≥18 years of age) including major depressive disorder, substance abuse, and related disorder, post-traumatic stress disorder, obsessive-compulsive disorder, bipolar disorder, panic disorder, schizophrenia, and generalized anxiety disorder were considered. Only those studies were considered that reported from any country within South Asia (i.e., Afghanistan, Bangladesh, Bhutan, India, Maldives, Nepal, Pakistan, and Sri Lanka). To obtain precise estimates of prevalence considered sufficiently powered studies with a minimum sample size of 250. Data regarding any specialized population (for example, depression among patients with a primary diagnosis of heart failure, cancer, stroke, etc.) was not included in this study. Only those studies were selected that were published between 2009 and 2019 to inform our review with the latest evidence base published from the South Asian region. Only original cross-sectional investigations or baseline data of longitudinal cohort studies were considered; which applied census or either probabilistic or non-probabilistic epidemiological procedures to ascertain prevalence CMDs. Essentially, we used cross-sectional data to extract point prevalence estimates. While from prospective studies, we extracted point or period prevalence estimates and lifetime prevalence estimates from most recent publications from the cohort.

### Data Extraction and Risk of Bias Assessment

All the data were extracted independently by three teams of reviewers using manual data extraction forms, and any disagreement among the reviewers was resolved through discussion with a senior author (SN). The last name of the first author, year of publication, study design, setting, geographical scope, educational and income level, country of study, percentage of male participants, and characteristics of study participants were extracted. Moreover, the data regarding the psychiatric disorder studied, mode and type of diagnostic interview, screening instruments, cut-off scores of the screening instruments, sampling techniques, sample size, response rates, and the number of participants with psychiatric and comorbid medical disorders was also tabulated.

Three teams of reviewers (AC, SK, NA, SS, NJ, and RA) assessed the quality of the studies independently without blinding to authorship or journal. At this stage, discrepancies in decision-making were resolved by discussion in conjunction with the senior author (SN). The risk of bias across studies was evaluated by using a modified version of the Newcastle Ottawa Scale (14), across the following matrices: representativeness of the study population, appropriate sample size, percentage of non-respondents, quality of tools used for the ascertainment of mental disorder, quality of reporting of descriptive statistics, informed consent, and reporting of appropriate ethical approval procedures.

### Data Analysis

Descriptive statistics on the characteristics of the study and populations were reported. Comprehensive meta-analysis software was used to run a series of meta-analyses to pool prevalence of specific mental disorders, using random-effects analysis (15). Due to anticipated heterogeneity in data owing to the use of different classification systems and psychometric scales to assess mental disorders, we employed random effects for meta-analysis throughout (16). Sensitivity analyses were conducted by excluding individual studies individually to ascertain their effects on the pooled prevalence. Begg’s funnel plot and Egger’s regression statistics (significant at P < 0.10) were used in evaluating publication bias (17), which was adjusted for by using Duvall & Tweedie’s Trim and Fill method (18). To ensure an appropriate statistical power, subgroup analyses were conducted for subgroups reported in more than four studies per outcome and meta-regression to identify significant moderators of prevalence when there were more than ten studies (19).

## Results

**Figure 1:**
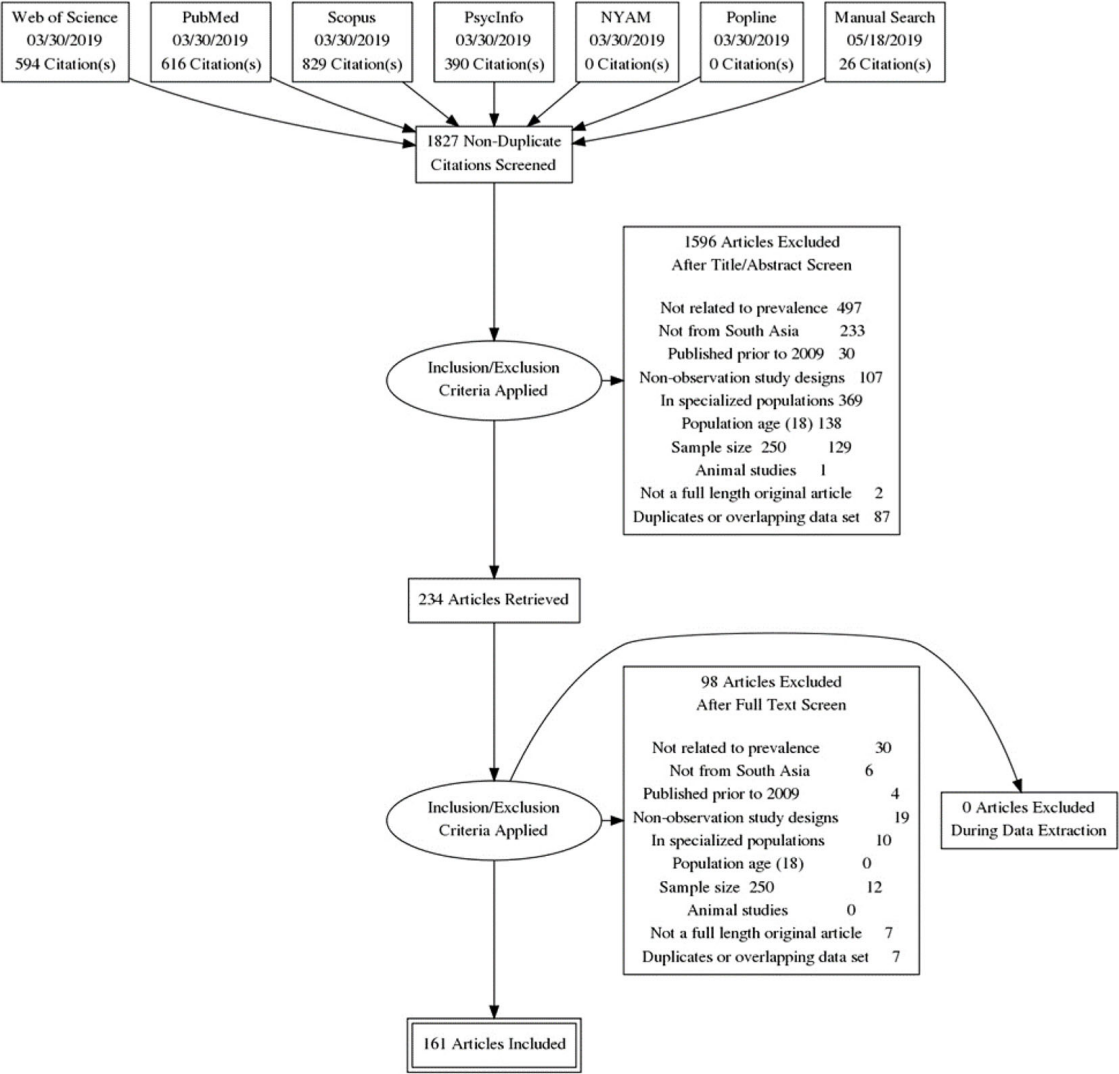
PRISMA flow diagram.

The initial literature search revealed 1,827 unique citations, among which 160 studies met the inclusion criteria of our meta-analytical studies. **Figure 1** elaborates the screening and selection process in the PRISMA flow diagram and **Supplementary Table 1** provides citations of all included studies. Among the included studies, only four had a longitudinal study design while the rest were cross-sectional studies. A majority of these studies were conducted in community settings (n = 133), followed by primary care centers (n = 11), tertiary care centers (n = 6), refugee settings (n = 2), and others (n = 8). A total of 67 studies were from urban settings, rural (n = 31), national (n = 8), provincial (n = 2), semi-urban (n = 4), and rest were conducted in mixed settings (n = 47). Participants in these studies were educated at undergraduate levels (n = 27), high school (n = 4), graduate (n = 4), and mixed (n = 97) while it was not mentioned in 28 studies. The highest proportion of these studies was conducted in India (n = 81) followed by Pakistan (n = 33), Nepal (n = 20), Sri Lanka (n = 12), Bangladesh (n = 8), while 6 were conducted across multiple countries. **Supplementary Table 2** summarizes the sociodemographic characteristics of participants included in the studies. A majority of the studies used scales to screen for mental disorders while 37 studies used diagnostic interviews based on DSM-IV (n = 12), ICD-10 (n = 12), ICD-11 (n = 1), MINI (n = 7), and composite international diagnostic interview (n = 5). The highest proportion of studies (n = 89) included face to face interviews, pencil based self-administered interviews (n = 65), and online surveys (n = 5). A variety of rating scales were used to assess these disorders.

We assessed the pooled prevalence for 17 different mental disorders over a period of 10 years. All the outcomes presented significant heterogeneity ranging from 0% to 99.79% for stimulant use and alcohol abuse, respectively. The prevalence of depressive symptoms was reported in 135 studies (*I* ^2^ = 99.53%) yielding a prevalence of 26.4% among 173,449 participants. Alcohol abuse was reported in 43 studies yielding a prevalence of 12.9% (8.8%–18.6%, *I* ^2^ = 99.79%, n = 107893); anxiety 25.8% (19.4% to 33.5%, *I* ^2^ = 99.57%, n = 70,058); tobacco smoking 18.6% (14.3% to 24%, *I* ^2^ = 99.58%, n = 84965); PTSD 17.2% (11% to 25.9%, *I* ^2^ = 99.55%, n = 42298); mixed anxiety and depression 28.4% (13.9% to 49.3%, *I* ^2^ = 99.41%, n = 11102); suicidal behaviors 6.4% (3.1% to 12.4%, *I* ^2^ = 99.41%, n = 25043); misuse of opiates 0.8% (0.2% to 2.5%, *I* ^2^ = 99.06%, n = 37304); tobacco chewing 21.0% (14.0% to 30.3%, *I* ^2^ = 98.49%, n = 10586); use of cannabis 3.4% (1.5% to 7.3%, *I* ^2^ = 97.48%, n = 10977); GAD 2.9% (0.3% to 26.5%, *I* ^2^ = 99.57%, n = 70058); bipolar disorder 0.6% (0.3% to 1.0%, *I* ^2^ = 78.21%, n = 7197); IV drug abuse 2.5% (0.1% to 32.1%, *I* ^2^ = 99.72%, n = 15049); Panic disorder 0.01% (0.05% to 0.3%, *I* ^2^ = 95.43%, n = 28087); stimulant use 0.9% (0.5% to 1.6%, *I* ^2^ = 0%, n = 1414); OCD 1.6% (0.4% to 5.5%, *I* ^2^ = 96.57%, n = 8784) and phobic disorders 1.8% (0.4% to 7.1%, *I* ^2^ = 98.16%, n = 27754). **Supplementary Figures 1–12** represent the forest plots for the above-mentioned disorders.

A meta-regression was conducted which revealed a significant geographical variation in the prevalence of CMDs, explaining 15% of the variation in total between-study variance. Taking Bangladesh as a reference point, the highest burden of mental illnesses was reported in Pakistan (B = 1.96). Nepal and Sri Lanka also reported a higher prevalence than Bangladesh. Moreover, no significant differences in the prevalence of CMDs were reported between Bangladesh and India. **Table 1** represents the findings of pooled prevalence for mental disorders in South Asia. **Table 2** represents the findings from Meta-regression analysis exhibiting South Asian countries as moderators of pooled prevalence of mental disorders in South Asia. Further meta-regression analyses did not reveal any association between prevalence estimates of all mental disorders with the year of publication (B = 0.04, P = 0.24, R^2^ analog = 0%). A total of 373 studies reported the percentage of males or female participants. Meta-regression analysis revealed that the percentage of males in the study explained 3% of the variation in heterogeneity (B = −0.02, P ≤ 0.001). These meta-regression plots are presented as **Supplementary Figures 1 and 2**.

**Table 1:** Pooled prevalence of mental disorders in South Asia.

Outcome	Pooled prevalence (95% CI)	Data points	Sample size	I^2^	Q	P
Any disorder*	14.2% (12.9% to 15.7%)	394	8,63,657	99.67%	100,099.20	<0.001
Depression	26.4% (23.6% to 29.4%)	135	173,449	99.53%	28,447	<0.001
Alcohol abuse	12.9% (8.8%–18.6%)	43	107,893	99.79%	20,683	<0.001
Anxiety	25.8% (19.4% to 33.5%)	36	70,058	99.57%	8,038.08	<0.001
Tobacco smoking	18.6% (14.3% to 24.0%)	34	84,965	99.58%	7,934.68	<0.001
PTSD	17.2% (11.0% to 25.9%)	21	42,298	99.55%	4,457.19	<0.001
Mixed anxiety and depression	28.4% (13.9% to 49.3%)	13	11,102	99.41%	2,043.01	<0.001
Suicidal behaviors	6.4% (3.1% to 12.4%)	13	25,043	99.41%	2,041	<0.001
Opiates	0.8% (0.2% to 2.5%)	12	37,304	99.06%	1,175.12	<0.001
Tobacco chewing	21.0% (14.0% to 3.03%)	10	10,586	98.49%	852.95	<0.001
Cannabis	3.4% (1.5% to 7.3%)	9	10,977	97.48%	317.52	<0.001
GAD	2.9% (0.3% to 26.5%)	5	31,682	99.77%	1,698.73	<0.001
Bipolar	0.6% (0.3% to 0.01%)	4	7,197	78.21%	13.77	0.003
IV Drug abuse	2.5% (0.1% to 32.1%)	4	15,049	99.72%	1,062.44	<0.001
Panic disorder	1.3% (0.5% to 3.4%)	4	28,087	95.43%	65.67	<0.001
Stimulants	0.9% (0.5% to 1.6%)	4	1,414	0%	1.09	0.78
OCD	1.6% (0.4% to 5.5%)	3	8,784	96.57%	58.29	<0.001
Phobias	1.8% (0.4% to 7.1%)	3	27,754	98.16%	108.88	<0.001

*Pooled estimate after adjusting for publication bias = 11.31% (10.05% to 12.69%).

**Table 2:** Meta-regression analysis exhibiting pooled prevalence of mental disorders in South Asian countries.

Covariate	Coefficient	Confidence Interval		P-value	Heterogeneity
		95% Lower	95% Upper		
Intercept	−2.2725	−2.9563	−1.5887	<0.001	
India	0.1364	−0.5936	0.8664	0.7143	Q = 54.84, df = 4, p ≤ 0.001
Nepal	0.9301	0.1205	1.7396	0.0243	Q = 54.84, df = 4, p ≤ 0.001
Pakistan	1.9675	1.1501	2.7848	<0.001	Q = 54.84, df = 4, p ≤ 0.001
Sri Lanka	0.9745	0.0414	1.9076	0.0407	Q = 54.84, df = 4, p ≤ 0.001

R square = 15%.

Subgroup analyses were conducted for several factors, showing large variations in prevalence estimates across different subgroups (**Table 3**). Studies employing diagnostic interviews presented a significantly lower prevalence rate of 5.22% when compared with questionnaire-based surveys (19.14%). Studies conducted in healthcare settings reported a much higher prevalence rate of CMDs than its counterparts conducted in the community or refugee settings. Studies employing random sampling procedures than non-random and having cross-sectional study designs reported lower prevalence rates.

**Table 3:** Subgroup analyses presenting several factors associated with the prevalence of CMDs in included in studies.

Group	Pooled prevalence	Lower limit	Upper limit	Q-value	df (Q)	P-value
**Method for identification of CMD**						
Diagnostic	5.22%	4.27%	6.37%	139.23	1.00	<0.001
Questionnaire	19.14%	17.38%	21.02%			
**Study setting**						
Community	13.05%	11.74%	14.49%	31.71	3.00	<0.001
Healthcare setting	29.01%	21.25%	38.24%			
Other	26.53%	17.38%	38.26%			
Refugee Settings	7.19%	3.19%	15.40%			
**Sampling Method**						
Non-random	19.0%	16.4%	21.9%	26.18	1.00	<0.001
Random	11/4%	10%	12.9%			
**Study design**						
Cross-sectional	13.93%	12.61%	15.35%	7.62	1.00	0.01
Longitudinal	30.52%	17.91%	46.94%			
**Background of participants**						
Mixed	14.37%	12.04%	17.06%	56.40	5.00	<0.001
National	18.18%	12.58%	25.53%			
Provincial	1.91%	1.03%	3.51%			
Rural	14.12%	10.96%	18.00%			
Semi-urban	36.58%	13.84%	67.43%			
Urban	17.47%	15.05%	20.18%			

**Figure 2:**
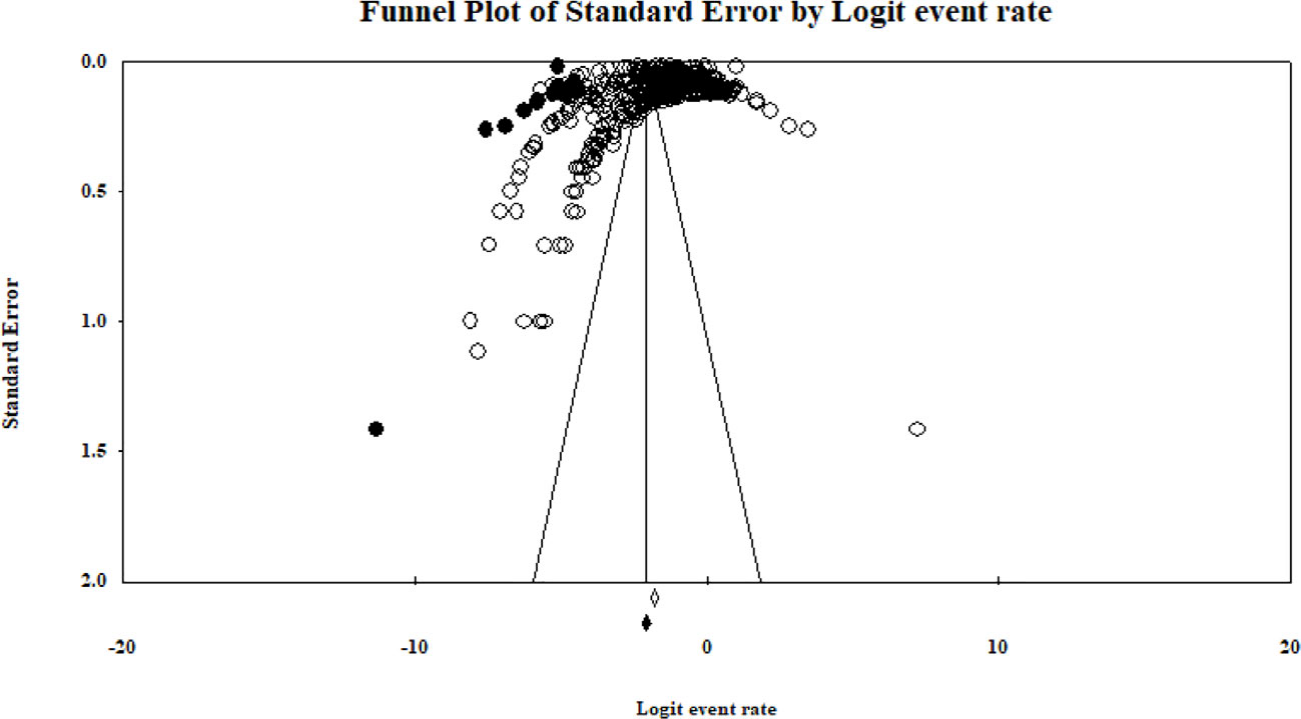
Funnel plot exhibiting publication bias in the studies.

In the risk of bias assessment, most studies (60%, n = 96) scored eight and above, which informs a low risk of bias across the recruited studies. Furthermore, a funnel plot of the standard error by logit event rate among studies showed publication bias in the literature, with a significant Egger’s regression statistic (Intercept = −3.14, P < 0.01). **Figure 2** presents the funnel plot showing studies currently present and imputed in the analysis. The pooled estimate for prevalence was adjusted by imputing 32 studies to the left of the mean, yielding an adjusted prevalence of 11.31% (10.05% to 12.69%).

The quality of the studies was measured across nine matrices: representativeness of the population, adequacy of sample size, reporting of characteristics of non-respondents, use of a commonly employed tool to ascertain psychiatric disorder, reporting of reliability measure and valid measure of scales, reporting of descriptive statistics, reporting of the method of informed consent and ethical approval. According to it, only 111 of the studies surveyed among populations that were representative of their setting, 122 reported ethical approval status, 126 characteristics of respondents, use of commonly employed methods for the ascertainment of mental disorder (n = 132), use of reliable (n = 136) and valid tool (n = 136), reporting informed consent procedures (n = 145), descriptive statistics (n = 146), and adequate sample size (n = 158). Overall, a small proportion of studies (n = 16) reported < 5 of these matrices, 6–8 (n = 90), and 56 scored positively on all of these criteria.

## Discussion

### Summary

South Asian countries have a high disease burden of psychiatric illnesses. Our study showed a prevalence of 14.2% (12.9% to 15.7%) for CMDs. This is higher compared to the worldwide-pooled prevalence of mental disorders was 13.4% (20). Our findings show the substantial public health burden of psychiatric disorders in South Asian populations. These findings may have significance for the mental health professionals as well as health policymakers of South Asian nations.

### Comparison With Previous Literature

The total prevalence of depressive symptoms in South Asian countries was reported in 135 studies, yielding a prevalence of 26.4% (23.6% to 29.4%) among 173,449 participants which is significantly higher than a previous study presenting data from 30 countries (15, 16). Moreover, our study reported a 6.4% prevalence of suicidal behaviors in South Asia. Prevalence estimates in developing countries are similar to those in developed countries for: suicidal ideation (3.1% to 12.4% versus 3.0% to 15.9%, respectively), suicide plan (0.9% to 4.1% vs. 0.7% to 5.6%, respectively), and suicide attempt (0.7% to 4.7% vs. 0.5% to 5.0%, respectively) (17). The suicide rate varies greatly between countries with different development levels. For South Asia, the countries with more accessible suicide data, overall mortality, and cause of death data reported higher national suicide rates. Therefore, it is likely that the current rate of suicide can be attributed to inaccurate or under-reporting of suicidal behaviors in the region (18, 19).

In Southeast Asia, a high prevalence of tobacco (smoking and smokeless) use in the general population has been observed (21). This meta-analysis found a higher prevalence of chewing tobacco compared to tobacco smoking (21% vs. 18.6%) in the south Asian countries. These results were similar to the findings of a literature review from 1986 to 2010 showing a smoking prevalence (median) was highest in Central/Eastern Europe (37%), followed by Africa (29%), Central and South America (25%), and Asia (17.5%) (22). Unlike developing countries, South Asian countries lack an effective framework to facilitate the people who chew or smoke tobacco to quit or national-level strategies to control tobacco through policy approaches (23).

Among the illicit use of substances, alcohol dependence is the most prevalent substance use disorder in South Asia, with a prevalence of 13%, which is consistent with previously conducted studies (24). While the most common drug use disorders in South Asia were cannabis dependence (3%) and IV drugs (2.5%), the rates of alcohol dependence are relatively lower than those reported in the Global Burden of Disease Study which reported 32.5% [95% uncertainty interval (UI) 30.0–35.2] of people globally were current drinkers (24). This disparity in findings may be due to different social structure in Muslim countries in South Asia which prohibit the use of alcohol (24). The higher prevalence of IV drug abuse (2.5%) in our analysis was particularly alarming. In comparison, the global estimated prevalence in 2015 was 0.33%, with an estimated 15.6 million people (95% UI 10·2–23·7 million) injected drugs (25). These results are alarming and explain the increase in diseases spread by the intravenous route in the South Asian countries (25). On the other hand, opiates and stimulant use were less common; 0.8% and 0.9%, respectively in our study.

### Explanation of Findings

The variation in the prevalence of mental disorders in different South Asian countries is speculated to be due to varying levels of psychosocial, cultural, and political stressors. Increasing inflation levels leading to economic stress, transitioning urban communities, inter-religious, sectarian, and ethnic violence, and war hysteria in countries such as India and Pakistan are recent examples of these stressors (26–28). In addition to these factors, the contribution of individual-level factors such as resilience, alexithymia, and “trait” markers of individuals to behavioral health and functionality needs to be considered (29–32).

### Recommendations for Future Practice

It is essential to improve the mental health services in South Asia, which will require further well-designed epidemiological and clinical research examining psychopathology of diverse population groups in this region. Moreover, public education and awareness campaigns on mental health conditions should be undertaken to ameliorate the substantial public health burden in South Asian countries. There should also be public awareness programs to convey the adverse long-term effects of tobacco, alcohol, and other illegal drugs. Also, treatment centers at the community level for drug addictions should be set up. In these discourses, evidence-based multipronged interventions found to be effective in other contexts (33–35) may inform the development and implementation of culturally appropriate mental health interventions in this region.

Legislations and mental health laws have significant shortcomings such as the lack of emphasis on human rights and community-based mental health care approaches. Moreover, there are major flaws in the implementation of these legislations. Therefore, mentally ill people continue to be vulnerable to various types of abuse and violation of their rights in the region (10). Additionally, there is a lack of comprehensive approach toward mental health policies as most countries lack the inclusion of substance use disorders in these policies (7, 10). In comparison to developed countries, common difficulties in this region include poor constitutional guarantees for proper health services, inadequate attention to civil and political rights, criminalization of symptoms of mental health symptoms such as suicide, and different cultural norms (10). Reform of mental health legislation plays a significant role along with increased trained manpower, improved resource allocation, and improved services (10). It is important to perform the needs assessment of the community for resource allocation and targeted community-based efforts.

## Limitations and Recommendations for Research

This meta-analysis has several limitations; therefore, its results should be interpreted with caution. Most of the included studies used screening instruments rather than diagnostic tools. Moreover, it was difficult to compare data from the selected studies due to differences in settings (clinic vs. community-based), different assessment tools, and the different scales used to determine the psychiatric disorders. Also, our review may have been subject to selection and publication bias as we were unable to contact the experts and collect unpublished materials or access any grey literature that may have met the criteria for this review.

Detailed analyses in our report primarily show that a high proportion of studies had poor study design, for instance, employing non-probabilistic sampling procedures, the use of screening instruments rather than diagnostic tools, and limited to a smaller study site. Although the present pooled analyses show high prevalence rates of different CMDs, we recommend future research employing diagnostic interviews to diagnose mental disorders and recruiting participants in multiple community settings with appropriate sample size calculation, to yield precise prevalence estimates.

## Conclusion

This systematic review and meta-analysis found a high prevalence of CMDs in South Asian countries. Among the eight nations in this region, Pakistan had the highest prevalence of CMDs. Also, the epidemiological burden of different diseases affects diverse population groups at varying levels, which need further research on how different risk factors contribute to such a high prevalence of mental disorders in South Asia. This study provides cumulative baseline evidence on the prevalence of CMDs and informs an urgent need for effective policymaking and multipronged interventions that are culturally appropriate in the context of South Asia.

## Data Availability Statement

All datasets presented in this study are included in the article/**Supplementary Material**.

## Author Contributions

SN and AW conceived the idea of this review article. SN, AW, AC, SK, NA, RA, NJ, and SS extracted and analyzed data, prepared tables, and wrote the manuscript. SN was responsible for the supervision of this project. SN and AC equally contributed to this work.

## Conflict of Interest

The authors declare that the research was conducted in the absence of any commercial or financial relationships that could be construed as a potential conflict of interest.
