# Corrigendum: Prevalence of Common Mental Disorders in South Asia: A Systematic Review and Meta-Regression Analysis

**DOI:** 10.3389/fpsyt.2020.602062

**Published:** 2020-11-20

**Authors:** Sadiq Naveed, Ahmed Waqas, Amna Mohyud Din Chaudhary, Sham Kumar, Noureen Abbas, Rizwan Amin, Nida Jamil, Sidra Saleem

**Affiliations:** ^1^Department of Child Psychiatry, Kansas University Medical Center, Kansas City, KS, United States; ^2^Institute of Population Health, University of Liverpool, Liverpool, United Kingdom; ^3^Nishtar Medical University, Multan, Pakistan; ^4^Dow Medical College, Karachi, Pakistan; ^5^FMH College of Medicine and Dentistry, Lahore, Pakistan; ^6^King Edward Medical University, Lahore, Pakistan; ^7^Fatima Jinnah Medical University, Lahore, Pakistan

**Keywords:** psychiatric illness, South Asia, prevalence, epidemiology, common mental disorders

In the original article, there was a mistake in Table 1 **“Pooled prevalence of mental disorders in South Asia”** as published. **Prevalence estimates of five of the psychiatric disorders were wrongly formatted with misplaced decimal points**. The corrected Table 1 **“Pooled prevalence of mental disorders in South Asia”** appears below.

**Table 1:** Pooled prevalence of mental disorders in South Asia.

**Outcome**	**Pooled prevalence (95% CI)**	**Data points**	**Sample size**	***I^**2**^***	***Q***	***P***
Any disorder^*^	14.2% (12.9% to 15.7%)	394	8,63,657	99.67%	100099.20	<0.001
Depression	26.4% (23.6% to 29.4%)	135	173449	99.53%	28447	<0.001
Alcohol abuse	12.9% (8.8%−18.6%)	43	107893	99.79%	20683	<0.001
Anxiety	25.8% (19.4% to 33.5%)	36	70058	99.57%	8038.08	<0.001
Tobacco smoking	18.6% (14.3% to 24.0%)	34	84965	99.58%	7934.68	<0.001
PTSD	17.2% (11.0% to 25.9%)	21	42298	99.55%	4457.19	<0.001
Mixed anxiety and depression	28.4% (13.9% to 49.3%)	13	11102	99.41%	2043.01	<0.001
Suicidal behaviors	6.4% (3.1% to 12.4%)	13	25043	99.41%	2041	<0.001
Opiates	0.8% (0.2% to 2.5%)	12	37304	99.06%	1175.12	<0.001
Tobacco chewing	21.0% (14.0% to 30.3%)	10	10586	98.49%	852.95	<0.001
Cannabis	3.4% (1.5% to 7.3%)	9	10977	97.48%	317.52	<0.001
GAD	2.9% (0.3% to 26.5%)	5	31682	99.77%	1698.73	<0.001
Bipolar disorder	0.6% (0.3% to 1.0%)	4	7197	78.21%	13.77	0.003
IV Drug abuse	2.5% (0.1% to 32.1%)	4	15049	99.72%	1062.44	<0.001
Panic disorder	1.3% (0.5% to 3.4%)	4	28087	95.43%	65.67	<0.001
Stimulants	0.9% (0.5% to 1.6%)	4	1414	0%	1.09	0.78
OCD	1.6% (0.4% to 5.5%)	3	8784	96.57%	58.29	<0.001
Phobias	1.8% (0.4 % to 7.1%)	3	27754	98.16%	108.88	<0.001

* *Pooled estimate after adjusting for publication bias= 11.31% (10.05% to 12.69%)*.

In the original article, there was a mistake in Table 3 **“Subgroup analyses presenting several factors associated with the prevalence of CMDs in included studies”** as published. **Prevalence estimates for subgroups of sampling methods were wrongly formatted**. The corrected Table 3 **“Subgroup analyses presenting several factors associated with the prevalence of CMDs in included studies”** appears below.

**Table 3:** “Subgroup analyses presenting several factors associated with the prevalence of CMDs in included studies”.

**Group**	**Pooled prevalence**	**Lower limit**	**Upper limit**	***Q*-value**	**df (Q)**	***P*-value**
**Method for identification of CMD**						
Diagnostic	5.22%	4.27%	6.37%	139.23	1.00	<0.001
Questionnaire	19.14%	17.38%	21.02%			
**Study setting**						
Community	13.05%	11.74%	14.49%	31.71	3.00	<0.001
Healthcare setting	29.01%	21.25%	38.24%			
Other	26.53%	17.38%	38.26%			
Refugee Settings	7.19%	3.19%	15.40%			
**Sampling Method**						
Non-random	19.0%	16.4%	21.9%	26.18	1.00	<0.001
Random	11.4%	10%	12.9%			
**Study design**						
Cross-sectional	13.93%	12.61%	15.35%	7.62	1.00	0.01
Longitudinal	30.52%	17.91%	46.94%			
**Background of participants**						
Mixed	14.37%	12.04%	17.06%	56.40	5.00	<0.001
National	18.18%	12.58%	25.53%			
Provincial	1.91%	1.03%	3.51%			
Rural	14.12%	10.96%	18.00%			
Semi-urban	36.58%	13.84%	67.43%			
Urban	17.47%	15.05%	20.18%			

In the original article, there was an error. **Prevalence estimates for panic disorder was wrongly formatted with misplaced decimal points in the results section of abstract and main text**.

A correction has been made to the ***Research section***, ***Paragraph Number 2***:

We assessed the pooled prevalence for 17 different mental disorders over a period of 10 years. All the outcomes presented significant heterogeneity ranging from 0% to 99.79% for stimulant use and alcohol abuse, respectively. The prevalence of depressive symptoms was reported in 135 studies (*I*^2^ = 99.53%) yielding a prevalence of 26.4% among 173,449 participants. Alcohol abuse was reported in 43 studies yielding a prevalence of 12.9% (8.8%−18.6%, *I*^2^ = 99.79%, n = 107893); anxiety 25.8% (19.4% to 33.5%, *I*^2^ = 99.57%, n = 70,058); tobacco smoking 18.6% (14.3% to 24%, *I*^2^ = 99.58%, n = 84965); PTSD 17.2% (11% to 25.9%, *I*^2^ = 99.55%, n = 42298); mixed anxiety and depression 28.4% (13.9% to 49.3%, *I*^2^ = 99.41%, n = 11102); suicidal behaviors 6.4% (3.1% to 12.4%, *I*^2^ = 99.41%, n = 25043); misuse of opiates 0.8% (0.2% to 2.5%, *I*^2^ = 99.06%, n = 37304); tobacco chewing 21.0% (14.0% to 30.3%, *I*^2^ = 98.49%, n = 10586); use of cannabis 3.4% (1.5% to 7.3%, *I*^2^ = 97.48%, n = 10977); GAD 2.9% (0.3% to 26.5%, *I*^2^ = 99.57%, n = 70058); bipolar disorder 0.6% (0.3% to 1.0%, *I*^2^ = 78.21%, n = 7197); IV drug abuse 2.5% (0.1% to 32.1%, *I*^2^ = 99.72%, n = 15049); Panic disorder 1.3% (0.5% to 3.4%, *I*^2^ = 95.43%, n = 28087); stimulant use 0.9% (0.5% to 1.6%, *I*^2^ = 0%, n = 1414); OCD 1.6% (0.4% to 5.5%, *I*^2^ = 96.57%, n = 8784) and phobic disorders 1.8% (0.4% to 7.1%, *I*^2^ = 98.16%, n = 27754). Supplementary Figures 1–12 represent the forest plots for the above-mentioned disorders.

In the original article, there was an error. **Prevalence estimates for panic disorder was wrongly formatted with misplaced decimal points in the results section of abstract and main text**.

A correction has been made to the ***abstract:***

A prevalence of depressive symptoms was 26.4% among 173,449 participants, alcohol abuse was 12.9% (n = 107,893); anxiety 25.8% (n = 70,058); tobacco smoking 18.6% (n = 84,965); PTSD 17.2% (n = 42,298); mixed anxiety and depression 28.4% (n = 11,102); suicidal behaviors 6.4% (n = 25,043); misuse of opiates 0.8% (n = 37,304); tobacco chewing 21.0% (n = 10,586); use of cannabis 3.4% (n = 10,977); GAD 2.9% (n = 70,058); bipolar disorder 0.6% (n = 7,197); IV drug abuse 2.5% (n = 15,049); panic disorder 1.3% (n = 28,087); stimulant use 0.9% (n = 1,414); OCD 1.6% (n = 8,784) and phobic disorders 1.8% (n = 27,754).

The authors apologize for these errors and state that this does not change the scientific conclusions of the article in any way. The original article has been updated.

