# Supplementary material for: Prevalence of Common Mental Disorders in South Asia: A Systematic Review and Meta-Regression Analysis
Source: Front Psychiatry. 2020 Sep 2;11:573150. doi: 10.3389/fpsyt.2020.573150 (PMC7492672; doi:10.3389/fpsyt.2020.573150)
Supplement: Supplementary file 1 [file DataSheet_1.docx]

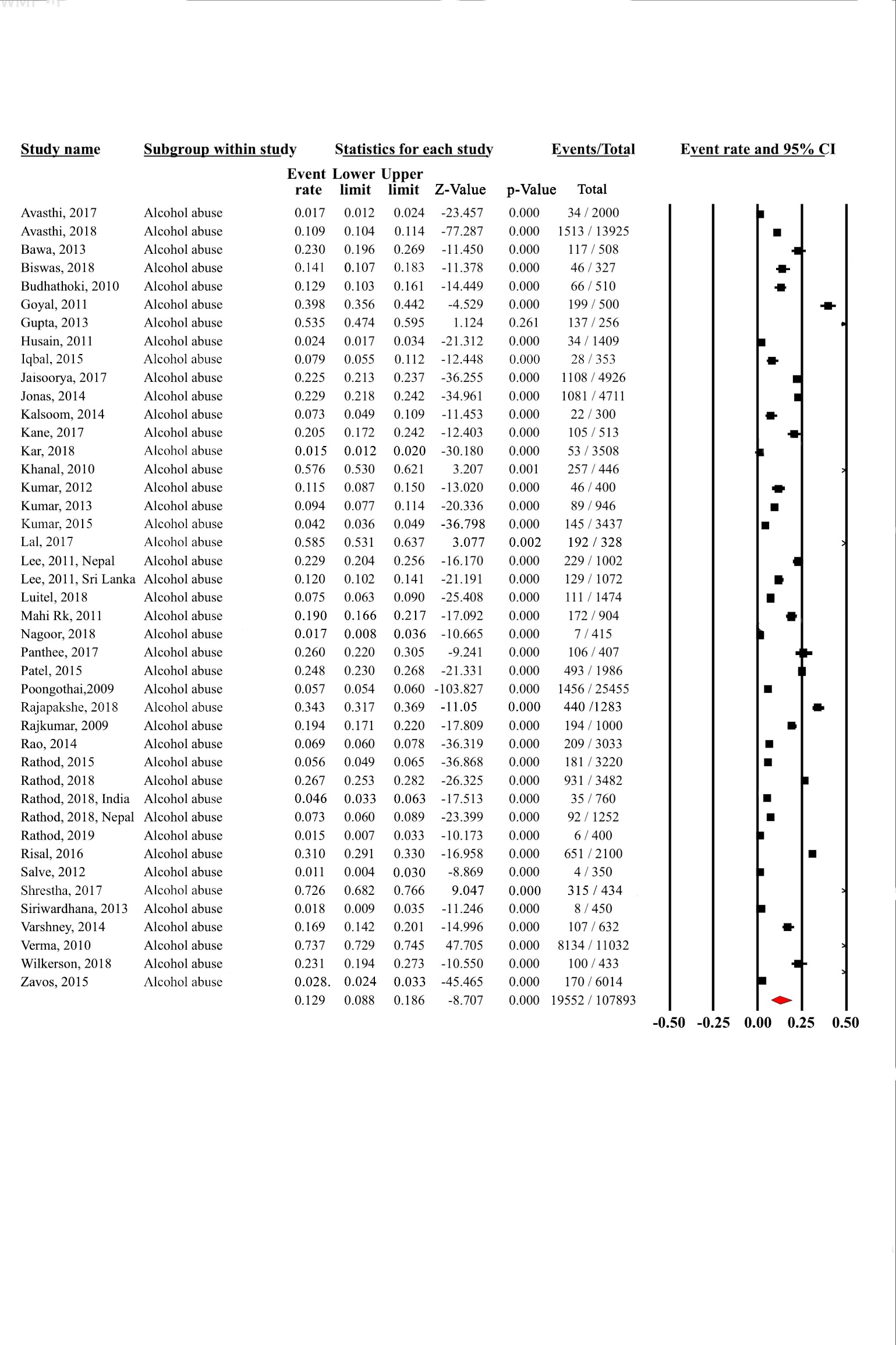


Figure 1: Forest Plot for Alcohol Use Disorder.


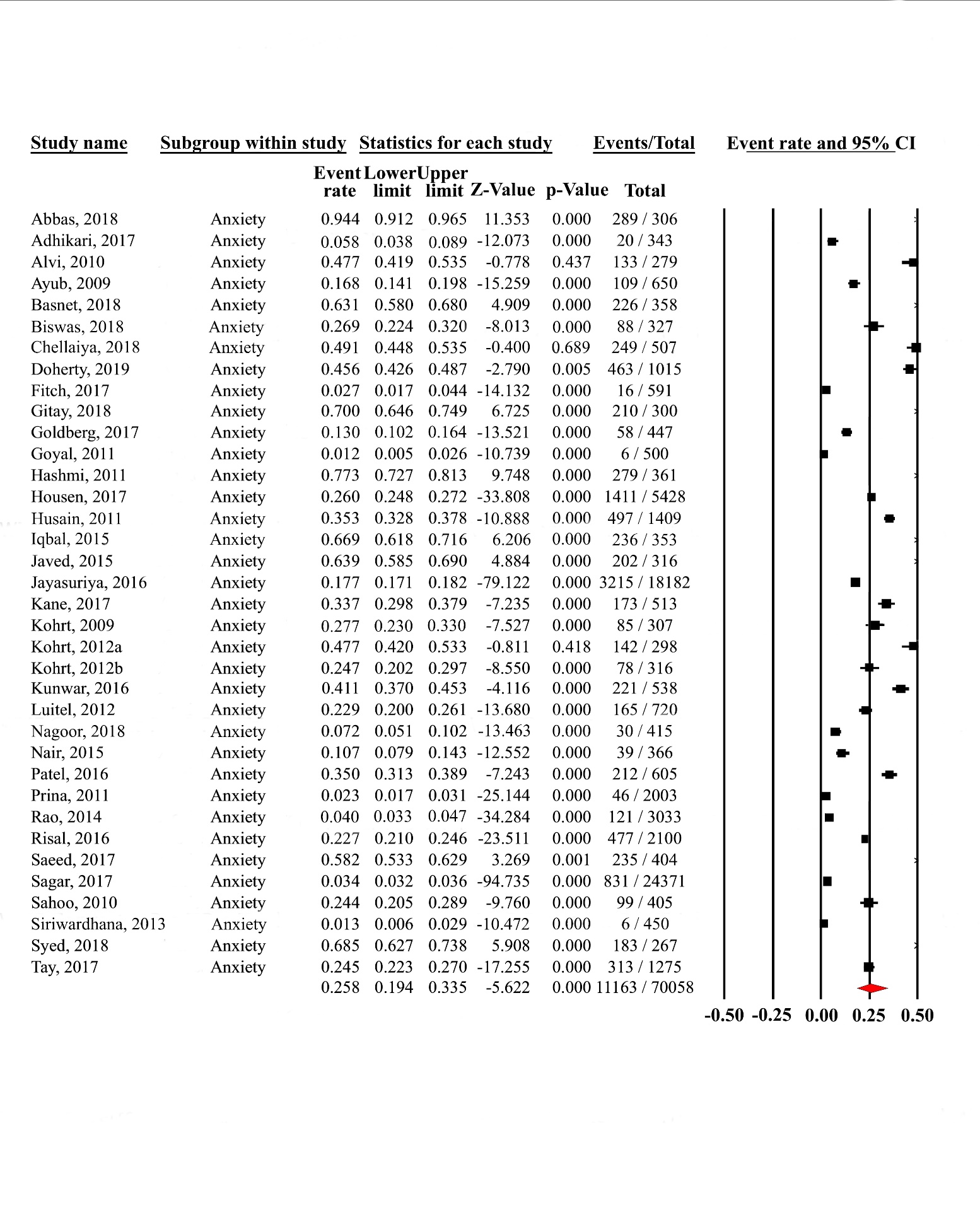


Figure 2: Forest Plot for Anxiety Disorder.

Figure 3: Forest Plot for Depressive Disorder.


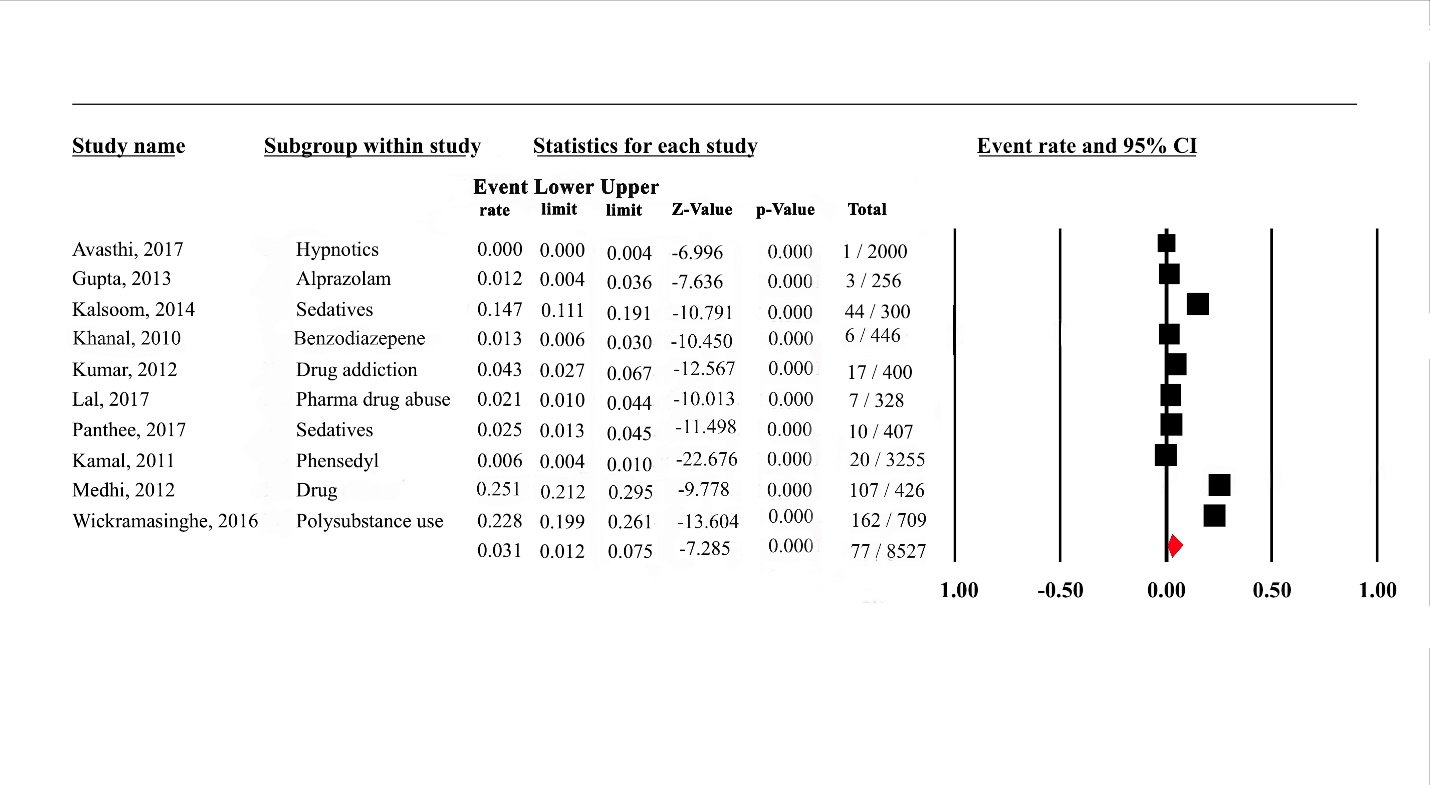


Figure 4: Forest Plot for Substance Use Disorder.


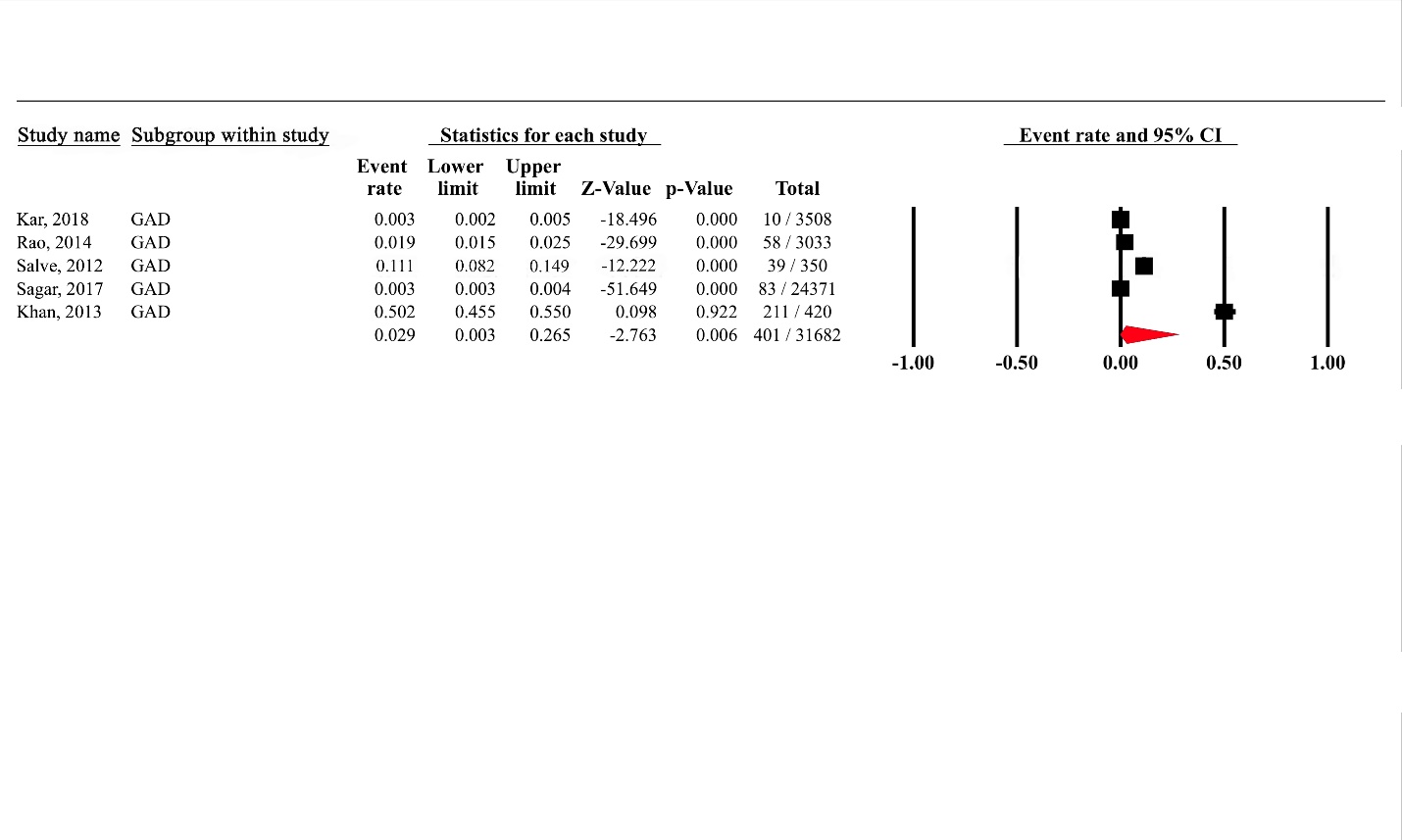


Figure 5: Forest Plot for GAD.


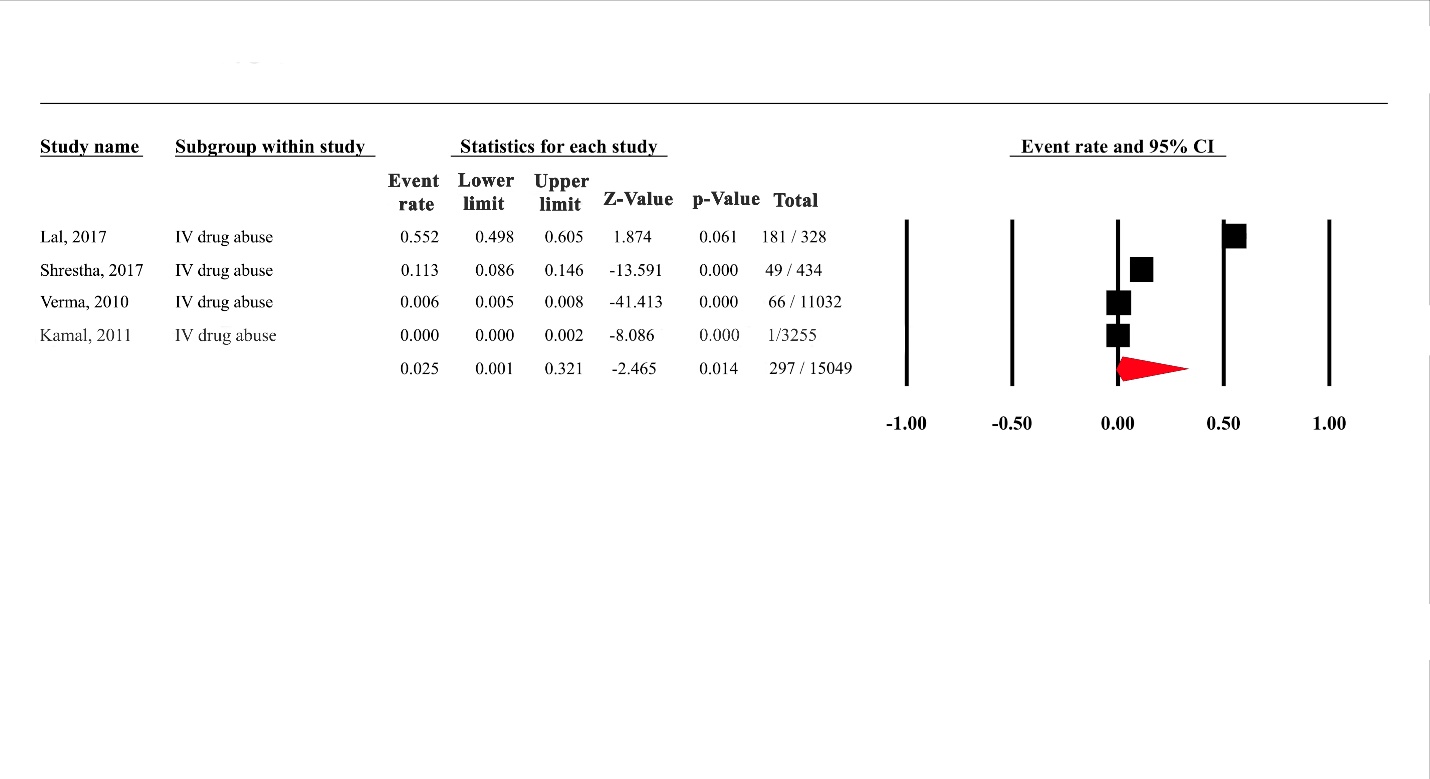


Figure 6: Forest Plot for Intravenous Drug Use.


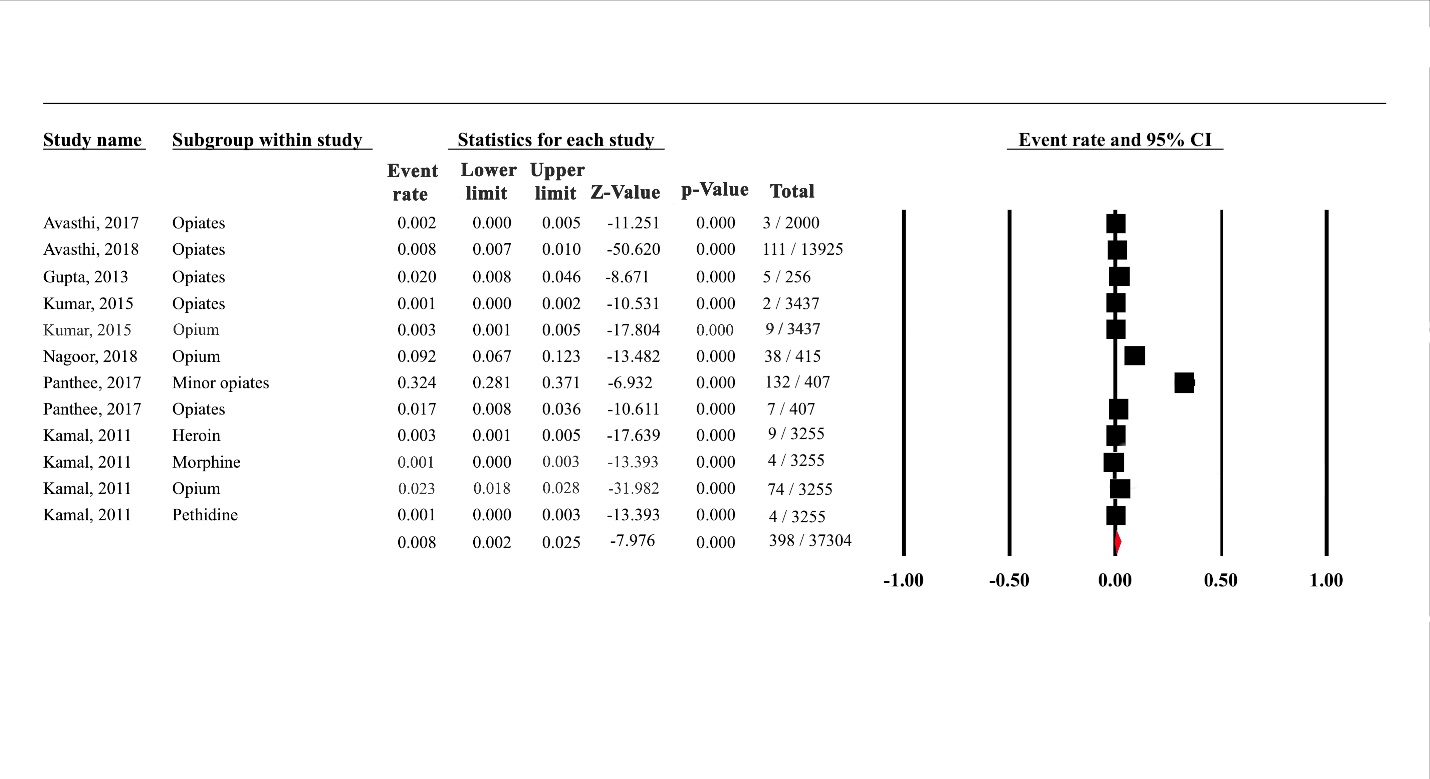


Figure 7: Forest Plot for Opioid use Disorder.


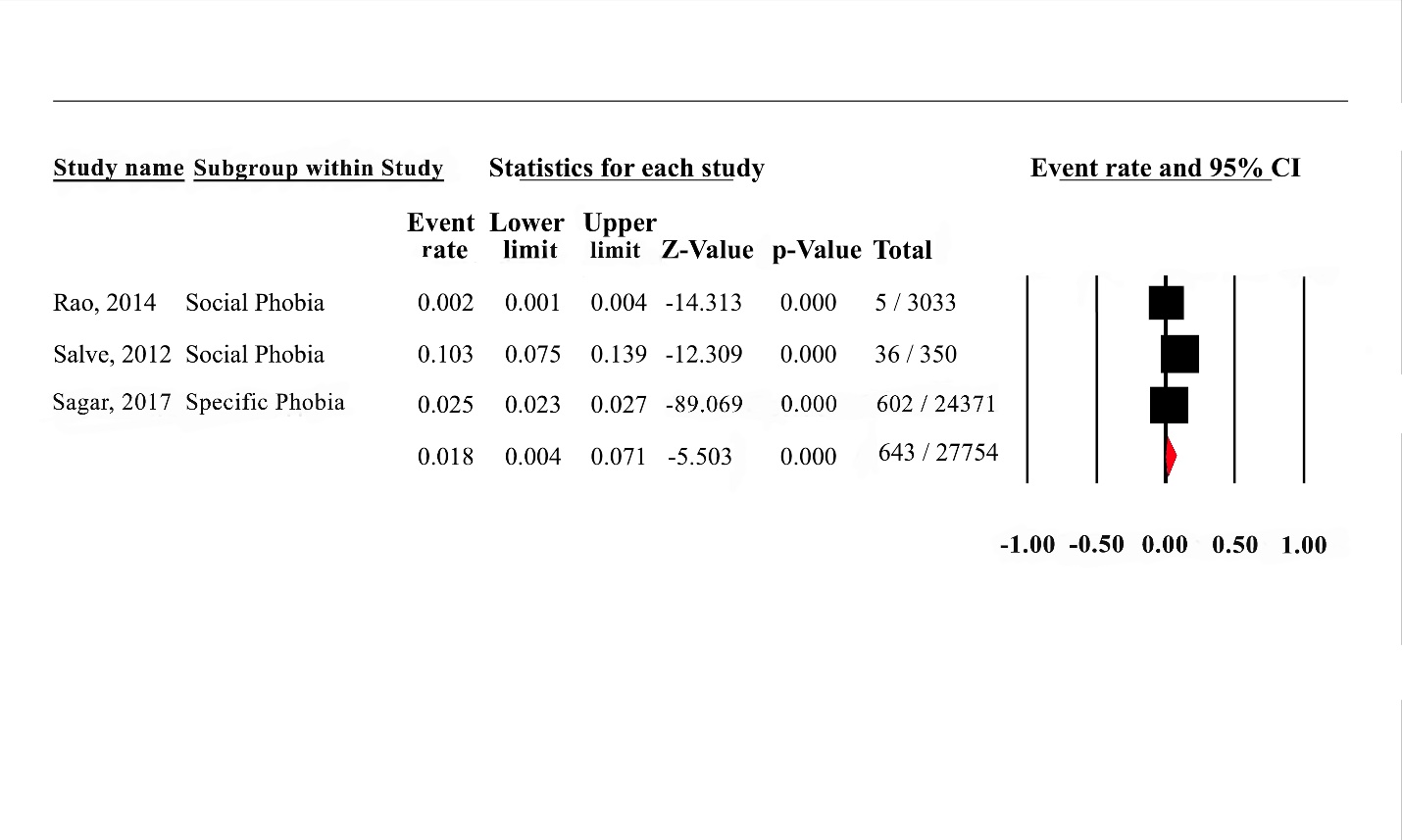


Figure 8: Forest Plot for Specific Phobia.


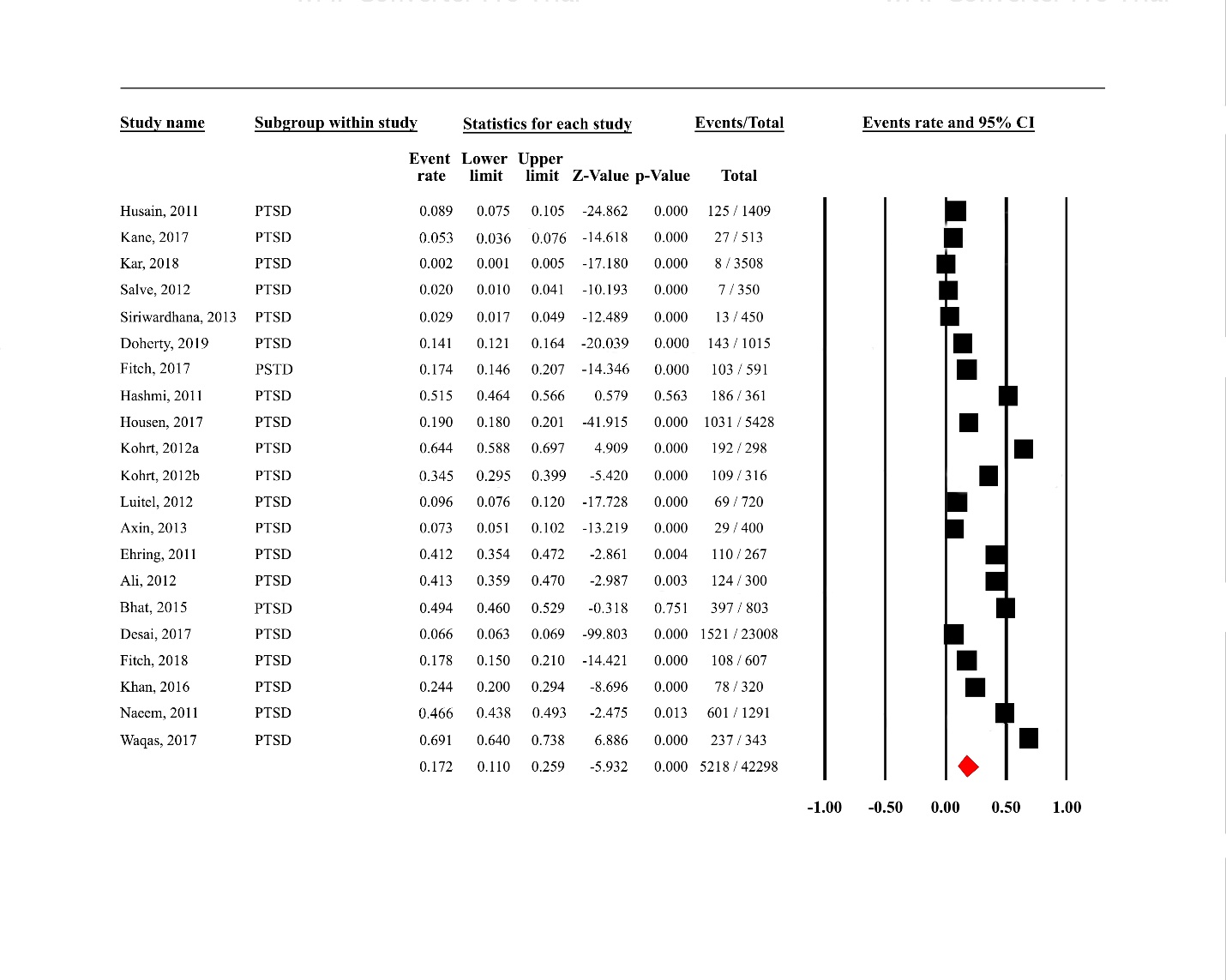


Figure 9: Forest Plot for Posttraumatic Stress Disorder.


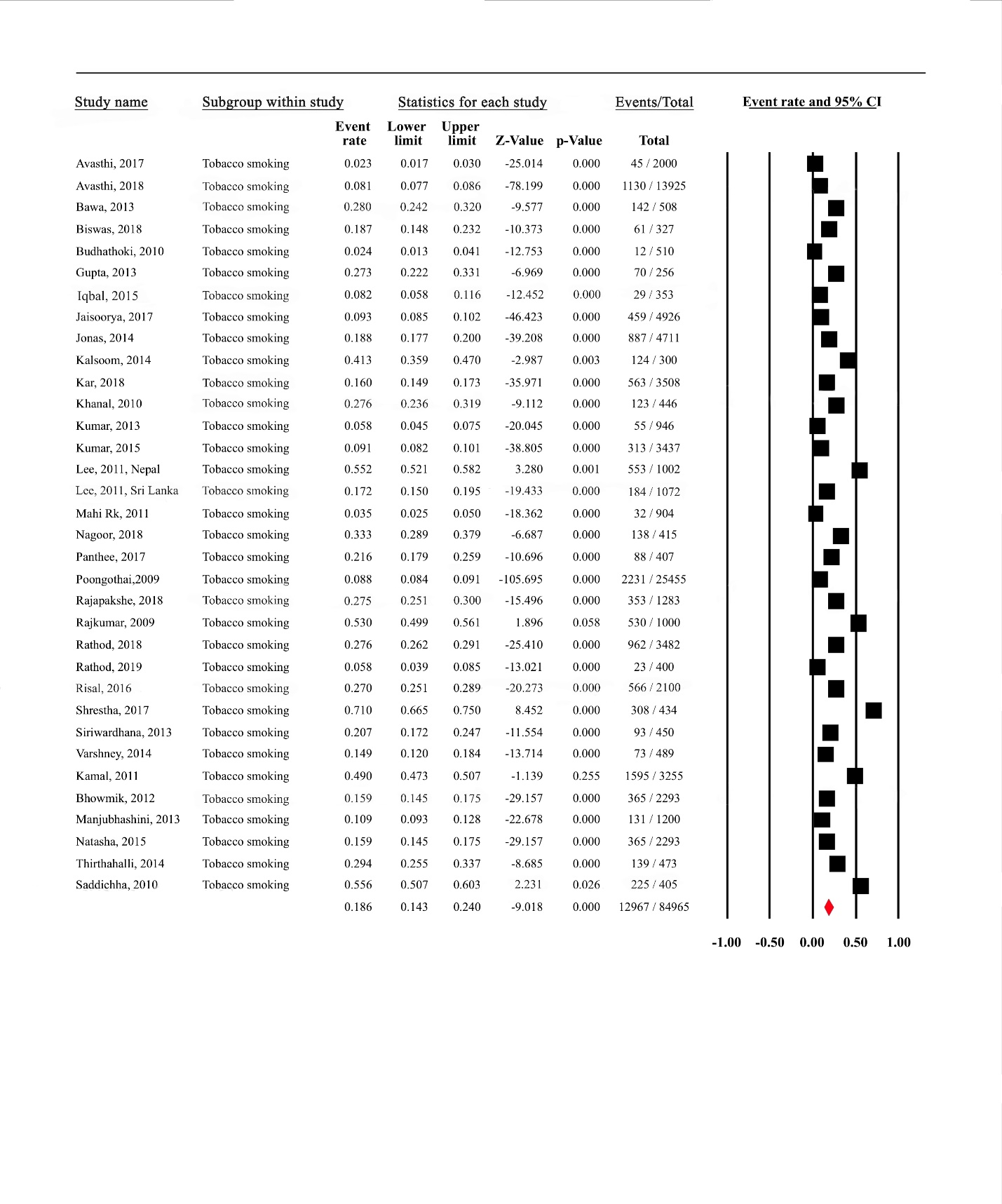


Figure 10: Forest Plot for Tobacco Use Disorder.


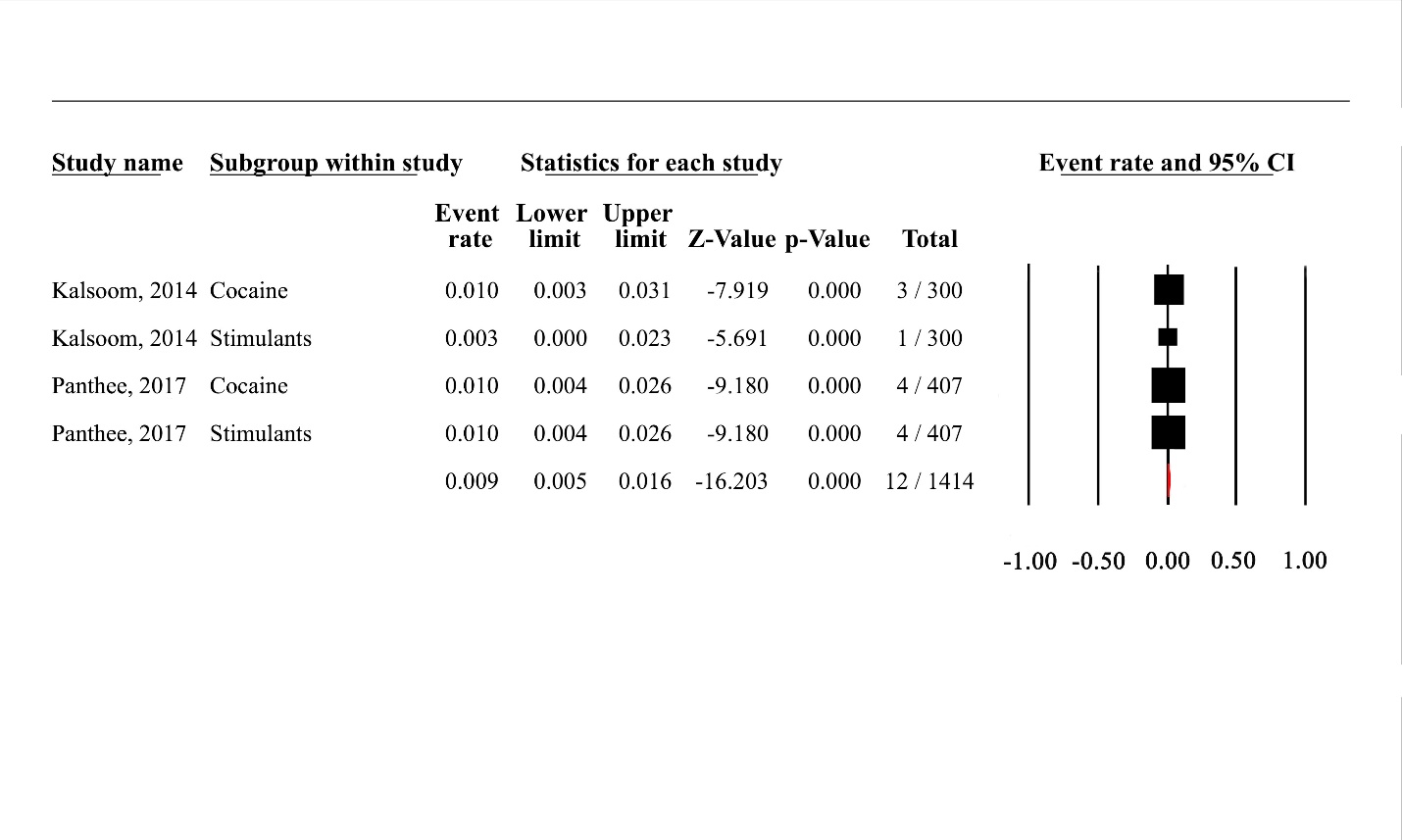


Figure 11: Forest Plot for Stimulant Disorder.


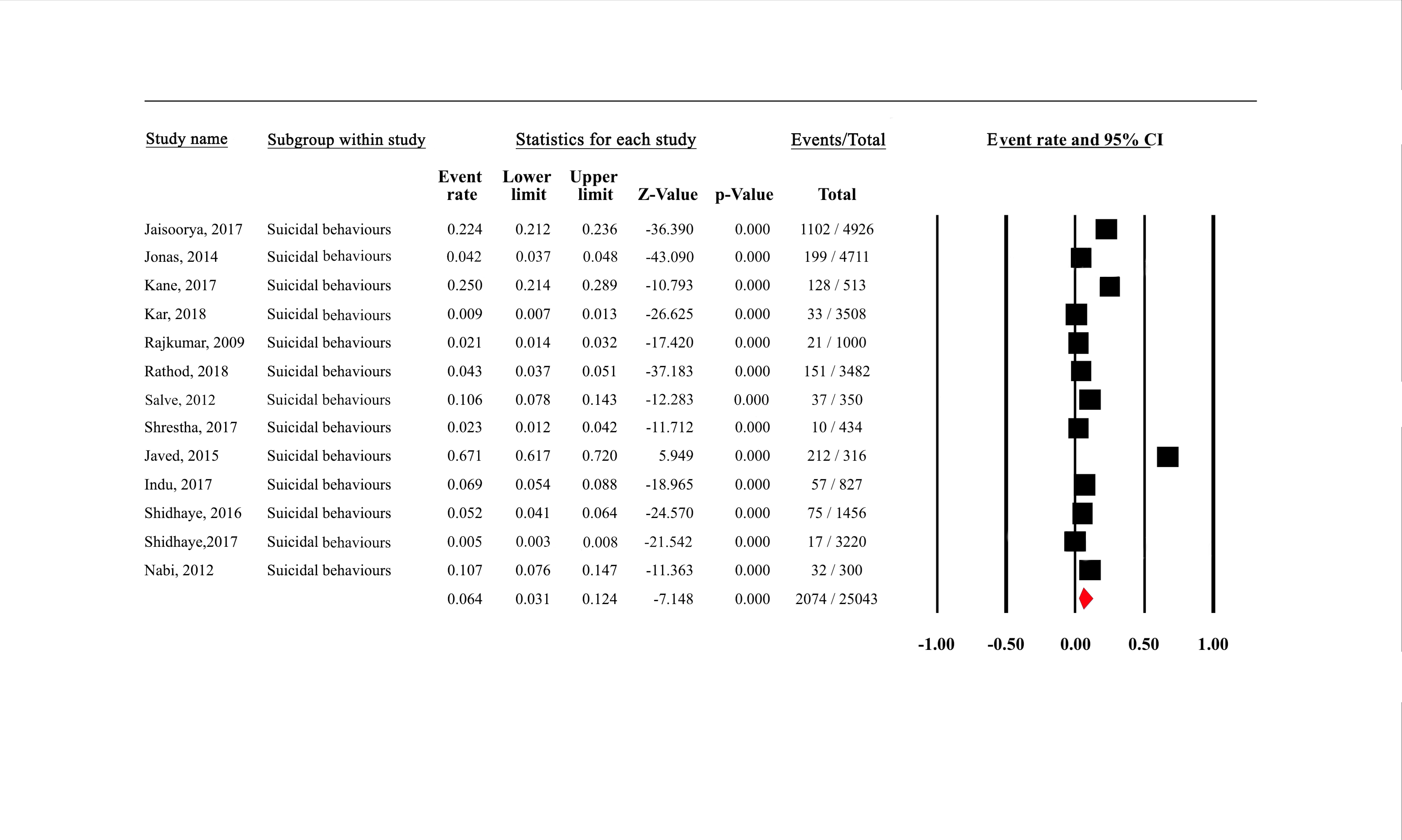


Figure 12: Forest Plot for Suicidal Behaviors.


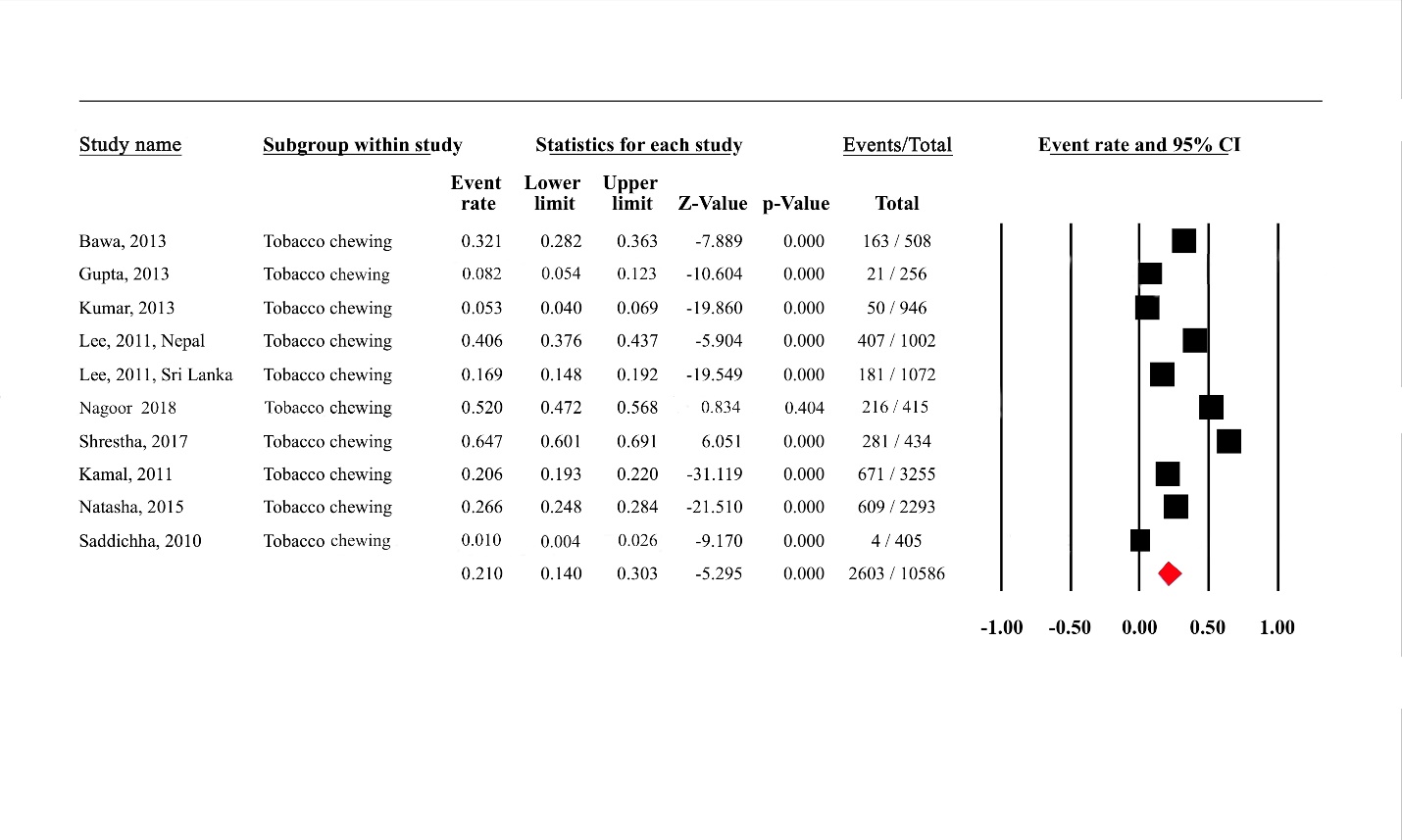


Figure 13: Forest Plot for Chewable Tobacco.


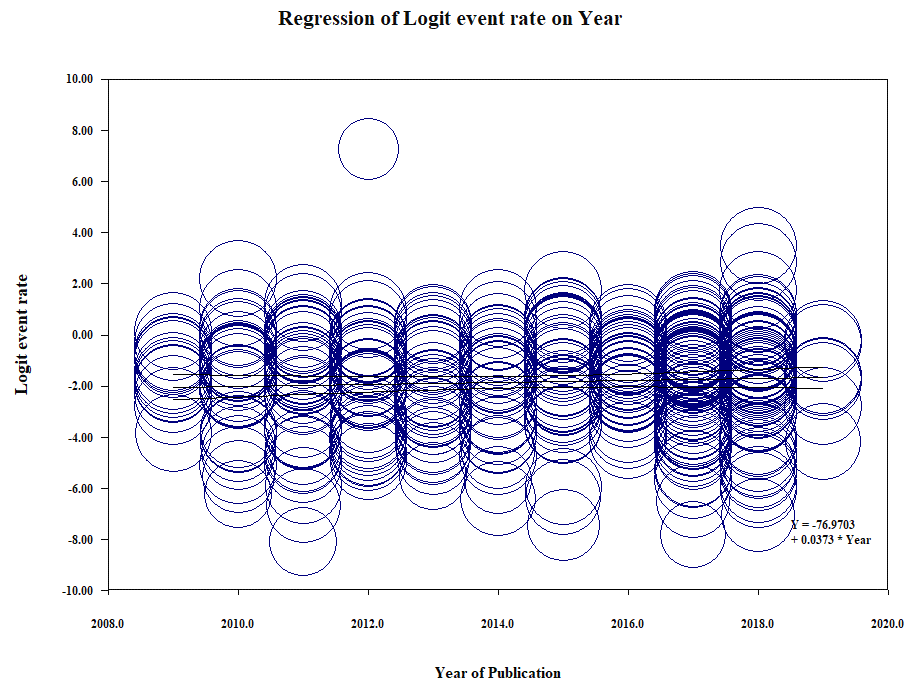


**Figure 14: Meta-regression exhibiting year of publication as a covariate of prevalence estimates.**


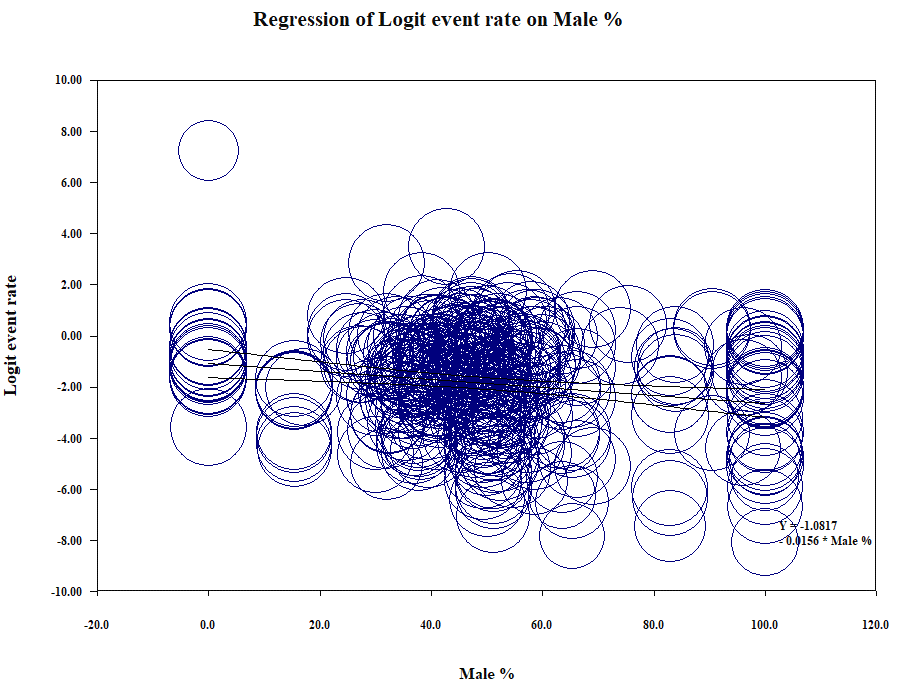


**Figure 15: Meta-regression exhibiting percentage of males as a covariate of prevalence estimates.**
