# Supplementary material for: Prevalence of Common Mental Disorders in South Asia: A Systematic Review and Meta-Regression Analysis
Source: Front Psychiatry. 2020 Sep 2;11:573150. doi: 10.3389/fpsyt.2020.573150 (PMC7492672; doi:10.3389/fpsyt.2020.573150)
Supplement: Supplementary file 2 [file Table_1.docx]

| **Study Authors, year of publication (Study ID)** | **Study design** | **Setting** | **Geographical scope** | **Country** | **Level of Education** | **Income group** | **Mean age** | **Percentage of males in the study** |
| --- | --- | --- | --- | --- | --- | --- | --- | --- |
| Abbas et al., 2015. (ID-9) | Cross-sectional | Community | Mixed | Pakistan | Undergraduate | Not specified | NA | 36.5 |
| Adhikari et al., 2017. (ID-25) | Cross-sectional | Community | Urban | Nepal | Undergraduate | Not specified | NA | 51 |
| Ahmed et al., 2016. (ID-47) | Cross-sectional | Community | Urban | India | Mixed | Not specified | 68.1 (6.37) | 48.1 |
| Abbas et al., 2018. (ID-52) | Cross-sectional | Community | Urban | India | Mixed | Not specified | 73.14 (9.002) | 32 |
| Akhtar et al., 2013. (ID-54) | Cross-sectional | Tertiary care center | Urban | India | Mixed | Not specified | 65.13 (4.92) | 75.4 |
| Albers et al., 2016. (ID-58) | Cross-sectional | Community | Mixed | India | Mixed | Not specified | 48.9 (8.2) | 52.9 |
| Khan et al., 2012. (ID-63) | Cross-sectional | Tertiary care center | Urban | Pakistan | Undergraduate | Not specified | 22 (5.50) | 0 |
| Ali et al., 2012. (ID-65) | Cross-sectional | Refugee setting | Urban | Pakistan | Mixed | Not specified | 37.8 (14) | 60.7 |
| Altaf et al., 2015. (ID-78) | Cross-sectional | Community | Urban | Pakistan | Mixed | Not specified | 42.73 (12.25) | 51.8 |
| Alvi et al., 2017. (ID-80) | Cross-sectional | Community | Mixed | Pakistan | Mixed | Not specified | 73 | NA |
| Alvi et al., 2010. (ID-83) | Cross-sectional | Community | Urban | Pakistan | Undergraduate | Not specified | 21.4 (1.41) | 27.6 |
| Archana et al., 2017. (ID-113) | Cross-sectional | Community | Rural | India | Mixed | Not specified | 48.67 (6.59) | 0 |
| Atif et al., 2016.  (ID-138) | Cross-sectional | Other | Urban | Pakistan | Graduate | Lower middle income | NA | 97.2 |
| Avasthi et al., 2017. (ID-142) | Cross-sectional | Community | Mixed | India | NA | Not specified | 35.86 | 65.4 |
| Avasthi et al., 2018. (ID-146) | Cross-sectional | Community | Mixed | India | NA | Not specified | 36.3 (12.7) | 66.2 |
| Ball et al., 2009. (ID-172) | Cross-sectional | Community | Mixed | Sri Lanka | Mixed | Not specified | NA | 46 |
| Baura et al., 2010. (ID-186) | Cross-sectional | Community | Rural | India | Mixed | Not specified | NA | 36 |
| Basnet et al., 2018. (ID-189) | Cross-sectional | Community | Mixed | Nepal | Mixed | Not specified | Median age=37,  Mean age=NA | 0 |
| Bawa et al., 2013. (ID-197) | Cross-sectional | Community | Urban | India | Mixed | Not specified | 36 (11) | 100 |
| Behera et al., 2016. (ID-201) | Cross-sectional | Community | Rural | India | Mixed | Not specified | 69.2 (7) | 43.5 |
| Bhamani et al., 2013. (ID-212) | Cross-sectional | Community | Urban | Pakistan | Mixed | Not specified | Men=67.8 (0.4),  Women=66.2 (0.3) | 53 |
| Bhandari et al., 2017. (ID-213) | Cross-sectional | Community | Urban | Nepal | Undergraduate | Not specified | 21.01 (2.18) | 45.4 |
| Bhat et al., 2015.  (ID-217) | Cross-sectional | Community | Urban | India | High school | Not specified | 19.9 | 58.8 |
| Bishwajit et al., 2017. (ID-229) | Cross-sectional | Community | National | Bangladesh, India, Nepal, Sri Lanka | Mixed | Not specified | Bangladesh=60.72 (9.6), India=59.94 (9.1), Nepal=60.75 (9), Sri Lanka=60 (9.08) | Bangladesh=51.2,  India=50.7,  Nepal= 51.2,  Sri Lanka=52.2 |
| Bishwajit et al., 2017. (ID-230) | Cross-sectional | Community | National | Bangladesh, India, Nepal, Sri Lanka | Mixed | Not specified | Bangladesh=39.66 (15.31),India=39.11 (15.32), Nepal=42.65 (16.58) | Bangladesh=42.8,  India=47.8,  Nepal=36.9 |
| Biswas et al., 2018. (ID-233) | Cross-sectional | Community | Urban | India | Undergraduate | Not specified | 20.9 (1.3) | 56.3 |
| Boralingaiah et al., 2012.  (ID-245) | Cross-sectional | Community | Urban | India | Mixed | Not specified | NA | 39.4 |
| Brinda et al., 2016. (ID-253) | Cross-sectional | Community | National | India | Mixed | Not specified | 71.2 (6.1) | 54.6 |
| Budhathoki et al., 2010. (ID-259) | Cross-sectional | Community | Urban | Nepal | Undergraduate | Not specified | NA | 63.5 |
| Bukhari et al., 2015. (ID-260) | Cross-sectional | Community | Urban | Pakistan | Graduate | Not specified | 21.70 (2.7) | 50.2 |
| Bushra et al., 2010. (ID-265) | Cross-sectional | Community | Urban | Pakistan | NA | Not specified | NA | NA |
| Buvneshkumar et al., 2018.  (ID-267) | Cross-sectional | Community | Rural | India | Mixed | Not specified | 68.14 (6.65) | 47.4 |
| Chaudhuri et al., 2017. (ID-301) | Cross-sectional | Community | Mixed | India | Mixed | Not specified | 33.17 (13.5) | 43.3 |
| Chellaiya et al., 2018. (ID-303) | Cross-sectional | Community | Urban | India | Undergraduate | Not specified | NA | 42.8 |
| Desai et al., 2017. (ID-370) | Longitudinal | Community | Mixed | India | NA | Not specified | NA | NA |
| Deswal et al., 2012. (ID-375) | Cross-sectional | Community | Urban | India | Mixed | Not specified | NA | 52.4 |
| Doherty et al., 2019. (ID-390) | Cross-sectional | Primary care center | Provincial | Sri Lanka | Mixed | Not specified | 53.2 (CI 56.2-65.1) | 47.8 |
| Kalsoom et al., 2014. (ID-404) | Cross-sectional | Community | Urban | Pakistan | Undergraduate | Not specified | 21.76 (1.16) | 58.6 |
| Ehring et al., 2011. (ID-409) | Cross-sectional | Other | Mixed | Pakistan | Mixed | Not specified | 28.93 (5.48) | 83.9 |
| Esie et al., 2019.  (ID-424) | Longitudinal | Community | Rural | Bangladesh | NA | Not specified | 24.4 (0.11) | 0 |
| Firdaus et al., 2014. (ID-449) | Cross-sectional | Community | Urban | India | NA | Not specified | NA | 34.78 |
| Fitch et al., 2018.  (ID-456) | Cross-sectional | Other | Mixed | Bangladesh | Mixed | Lower middle income | Garment workers= 27.9 (7.3), Non-garment workers=33.4 (9.7) | 0 |
| Ghayas et al., 2014. (ID-486) | Cross-sectional | Community | Urban | Pakistan | Undergraduate | Not specified | 21.36 (2.59) | 39.2 |
| Ghimire et al., 2018. (ID-488) | Cross-sectional | Tertiary care center | Mixed | Nepal | Mixed | Not specified | 68.5 (6.5) | 58.8 |
| Gitay et al., 2018.  (ID-494) | Cross-sectional | Community | Urban | Pakistan | Undergraduate | Not specified | NA | 38.3 |
| Goldberg et al., 2017. (ID-498) | Cross-sectional | Primary care center | Urban | Pakistan | NA | Not specified | 38.3 (13.3) | NA |
| Goswami et al., 2017. (ID-506) | Cross-sectional | Community | Rural | India | Mixed | Not specified | NA | 44.50% |
| Guerra et al., 2016. (ID-532) | Cross-sectional | Community | Mixed | India | Mixed | Not specified | Urban India=71.2, Rural India=72.5 | Urban India=42.4, Rural India=45.5 |
| Gupta et al., 2013. (ID-549) | Cross-sectional | Community | Urban | India | High school | Not specified | NA | 100 |
| Hashmi et al., 2011. (ID-572) | Cross-sectional | Community | Urban | Pakistan | Mixed | Not specified | 35 (13.8) | Azad Kashmir=39,  NWFP=48.5 |
| Housen et al., 2017. (ID-597) | Cross-sectional | Community | Mixed | India | Mixed | Not specified | 38.2 (5.4) | 35.4 |
| Husain et al., 2011. (ID-610) | Cross-sectional | Community | Urban | Sri Lanka | Mixed | Not specified | Currently displaced=32.74 (10.30), Resettled residents=40.13 (16.27), Long-term residents=40.24 (17.82) | 31.8 |
| Indu et al., 2017.  (ID-634) | Cross-sectional | Primary care center | Urban | India | NA | Not specified | 38 (11.1) | 40.6 |
| Iqbal et al., 2015.  (ID-640) | Cross-sectional | Community | Urban | India | Undergraduate | Not specified | Male=21.38 (1.71), Female=20.43 (1.37) | 41.1 |
| Jadoon et al., 2010. (ID-653) | Cross-sectional | Community | Urban | Pakistan | Undergraduate | Not specified | 20.66 (1.8) | 53.3 |
| Jaisoorya et al., 2017. (ID-663) | Cross-sectional | Community | Urban | India | Undergraduate | Not specified | 20.3 | 35.5 |
| Javed et al., 2015. (ID-682) | Cross-sectional | Tertiary care center | Urban | Pakistan | Mixed | Not specified | 37.75 (12.26) | 47.2 |
| Jonas et al., 2014.  (ID-704) | Cross-sectional | Community | Rural | India | Mixed | Not specified | 49.5 (13.4) | 46.5 |
| Kamal et al., 2011. (ID-726) | Cross-sectional | Community | NA | Bangladesh | Mixed | Not specified | 31.2 (11.2) | 100 |
| Kamble et al., 2009. (ID-728) | Cross-sectional | Primary care center | Rural | India | Mixed | Not specified | NA | 47 |
| Kar et al., 2018.  (ID-734) | Cross-sectional | Community | Provincial | India | Mixed | Not specified | NA | 51.2 |
| Kato et al., 2016.  (ID-749) | Cross-sectional | Community | NA | India | Mixed | Not specified | 36.06 (11.28) | 50 |
| Kausar et al., 2015. (ID-761) | Cross-sectional | Community | Urban | Pakistan | Mixed | Not specified | NA | NA |
| Khaltar et al., 2017. (ID-781) | Cross-sectional | Community | Semi-Urban | Sri Lanka | Mixed | Not specified | NA | 38.7 |
| Khan et al., 2016.  (ID-791) | Cross-sectional | Community | Urban | Pakistan | Undergraduate | Not specified | 20.94 (1.98) | 49.7 |
| Khan et al., 2013.  (ID-800) | Cross-sectional | Community | Urban | Pakistan | Mixed | Not specified | Male=33.1 (15.2),  Female=33.5 (13.7) | 58.6 |
| Khanal et al., 2010. (ID-812) | Cross-sectional | Community | Urban | Nepal | Undergraduate | Not specified | 21.2 (2.28) | 57.2 |
| Khanzada et al., 2015. (ID-815) | Cross-sectional | Community | Urban | Pakistan | Mixed | Not specified | NA | 52.04 |
| Kohli et al., 2013. (ID-830) | Cross-sectional | Primary care center | Rural | India | Mixed | Not specified | 31.73 (12) | 33 |
| Kohrt et al., 2009. (ID-834) | Cross-sectional | Community | Mixed | Nepal | NA | Not specified | NA | 60.6 |
| Kulkarni et al., 2014. (ID-854) | Cross-sectional | Community | National | India | Mixed | Not specified | 61.5 (9.01) | 51 |
| Kumar et al., 2012. (ID-860) | Cross-sectional | Community | Urban | India | Undergraduate | Not specified | NA | 54.3 |
| Kumar et al., 2015. (ID-867) | Cross-sectional | Community | Mixed | India | NA | Not specified | NA | 82.9 |
| Kumar et al., 2017. (ID-869) | Cross-sectional | Community | Urban | India | Undergraduate | Not specified | NA | 51.4 |
| Kunwar et al., 2016. (ID-876) | Cross-sectional | Community | Urban | Nepal | Undergraduate | Not specified | NA | 48 |
| Lal et al., 2017.  (ID-884) | Cross-sectional | Community | Mixed | India | Mixed | Not specified | NA | 90.5 |
| Lam et al., 2017.  (ID-885) | Cross-sectional | Primary care center | Semi-Urban | Nepal | Mixed | Not specified | 40.3 (16.4) | 41.6 |
| Lee et al., 2011.  (ID-906) | Cross-sectional | Community | National | Nepal, Sri Lanka | Mixed | Not specified | NA | Nepal=66.3,  Sri Lanka=35.9 |
| Luitel et al., 2018. (ID-925) | Cross-sectional | Primary care center | Mixed | Nepal | Mixed | Not specified | 39.4 | 34.2 |
| Luitel et al., 2012. (ID-927) | Cross-sectional | Community | Rural | Nepal | Mixed | Not specified | 37 (16.1) | 51 |
| Luni et al., 2009.  (ID-931) | Cross-sectional | Community | Rural | Pakistan | Mixed | Not specified | 33.2 (12.3) | 44.7 |
| Malhotra et al., 2010. (ID-955) | Cross-sectional | Community | Mixed | Sri Lanka | NA | Not specified | NA | 44.8 |
| Malik et al., 2014. (ID-960) | Cross-sectional | Community | Rural | India | NA | Not specified | 47.70 (16.11) | 17.1 |
| Mathias et al., 2015. (ID-990) | Cross-sectional | Community | Mixed | India | Mixed | Not specified | 39.4 | 50 |
| Medhi et al., 2012. (ID-1015) | Cross-sectional | Other | Mixed | India | Mixed | Not specified | Ever use drugs=26,  Never used drugs=25 | 0 |
| Mubeen et al., 2012. (ID-1062) | Cross-sectional | Community | Urban | Pakistan | Mixed | Not specified | 68.44 (7.59) | 73.9 |
| Swarnalatha et al., 2013.  (ID-1087) | Cross-sectional | Community | Rural | India | Mixed | Not specified | NA | 50 |
| Nabi et al., 2012.  (ID-1088) | Cross-sectional | Community | Urban | Pakistan | Graduate | Not specified | 32.6 (6.9) | 48.3 |
| Naeem et al., 2011. (ID-1092) | Cross-sectional | Community | Urban | Pakistan | Mixed | Not specified | Male= 34.66,  Female= 31.73 | 39.7 |
| Nagoor et al., 2018. (ID-1098) | Cross-sectional | Community | Rural | India | Mixed | Not specified | 67.2 (7.8) | 47.9 |
| Nair et al., 2015.  (ID-1111) | Cross-sectional | Community | Rural | India | NA | Not specified | NA | 46.4 |
| Natasha et al., 2015. (ID-1129) | Cross-sectional | Community | Rural | Bangladesh | Mixed | Not specified | 41.8 (13.8) | 36.7 |
| Padda et al., 2016. (ID-1204) | Cross-sectional | Community | Mixed | India | High School | Not specified | Median age=24,  Mean age=NA | 43 |
| Panthee et al., 2017. (ID-1216) | Cross-sectional | Community | Urban | Nepal | Undergraduate | Not specified | 22.2 (3.7) | 37.8 |
| Patel et al., 2016.  (ID-1230) | Cross-sectional | Community | Mixed | India | NA | High income | Students=21 (2.5), Homemakers=36.1 (6.3), Working women=35.2 (8) | 0 |
| Patel, 2016.  (ID-1231) | Cross-sectional | Community | Mixed | India | Mixed | Not specified | 31 (5.8) | 0 |
| Patel et al., 2015.  (ID-1232) | Cross-sectional | Community | Mixed | India | Mixed | Not specified | 29.2 (5.3) | 0 |
| Perveen et al., 2016.  (ID-1254) | Cross-sectional | Community | Urban | Pakistan | Undergraduate | Not specified | NA | 43.1 |
| Chawla et al., 2018. (ID-1260) | Cross-sectional | Community | Mixed | India | Mixed | Not specified | 67.4 (7.25) | 59.1 |
| Poongothai et al., 2009. (ID-1275) | Cross-sectional | Community | Urban | India | Mixed | Not specified | 39 (14) | 49.3 |
| Prina et al., 2011.  (ID-1294) | Cross-sectional | Community | National | India | NA | Not specified | NA | 30 (India) |
| Rajapakshe et al., 2018. (ID-1321) | Cross-sectional | Community | Urban | Sri Lanka | Mixed | Not specified | 66.15 (4.02) | 47.2 |
| Rajkumar et al., 2009. (ID-1326) | Cross-sectional | Community | Rural | India | Mixed | Not specified | 72.54 (5.87) | 45.4 |
| Rao et al., 2014.  (ID-1345) | Cross-sectional | Community | Rural | India | Mixed | Not specified | NA | 50 |
| Rathod et al., 2019. (ID 1348) | Cross-sectional | Community | Urban | India | Mixed | Not specified | NA | 38 |
| Rathod et al., 2018. (ID-1351) | Cross-sectional | Community | Mixed | Nepal | Mixed | Not specified | NA | 32.4 |
| Rathod et al., 2015. (ID-1352) | Cross-sectional | Community | Mixed | India | Mixed | Not specified | 40.2 (5.1) | 54.6 |
| Rathod et al., 2018. (ID-1353) | Cross-sectional | Primary care center | Mixed | India, Nepal | Mixed | Not specified | India 37,  Nepal 36 | India=49.2,  Nepal=35.1 |
| Risal et al., 2016.  (ID-1375) | Cross-sectional | Community | National | Nepal | NA | Not specified | 36.4 (12.8) | 41 |
| Saddichha et al., 2010. (ID-1413) | Cross-sectional | Community | Urban | India | Undergraduate | Not specified | 19.3 (2.8) | 100 |
| Saeed et al., 2017. (ID-1419) | Cross-sectional | Community | Urban | India | Graduate | Not specified | 19.3 (2.8) | 47.8 |
| Sagar et al., 2017. (ID-1425) | Cross-sectional | Community | Mixed | Pakistan | Mixed | Not specified | NA | 49.3 |
| Sahoo et al., 2010. (ID-1429) | Cross-sectional | Community | Urban | India | Undergraduate | Not specified | NA | 100 |
| Salve et al., 2012.  (ID-1434) | Cross-sectional | Community | Urban | India | NA | Not specified | NA | 15.4 |
| Senarath et al., 2014. (ID-1472) | Cross-sectional | Primary care center | Mixed | Sri Lanka | NA | Not specified | 43.2 (15.6) | 43.4 |
| Sengupta et al., 2015. (ID-1473) | Cross-sectional | Primary care center | Mixed | India | Mixed | Not specified | NA | 45.6 |
| Shidhaye et al., 2016. (ID-1523) | Cross-sectional | Community | Rural | India | Mixed | Not specified | 43.3 (15.5) | 52.3 |
| Shidhaye et al., 2017. (ID-1525) | Cross-sectional | Community | Mixed | India | Mixed | Not specified | 40.08 (15.4) | 54.6 |
| Parvin et al., 2018. (ID-1527) | Cross-sectional | Other  (Female garment workers) | Urban | Bangladesh | Mixed | Not specified | 27.4 (5.7) | 0 |
| Shrestha et al., 2017. (ID-1537) | Cross-sectional | Other  (Inmates in a prison in Nepal) | Mixed | Nepal | NA | Not specified | 35.7 (13.3) | NA |
| Simkhada et al., 2017. (ID-1555) | Cross-sectional | Community | Semi Urban | Nepal | Mixed | Not specified | 71.2 (8.38) | 45.3 |
| Singh et al., 2011. (ID-1559) | Cross-sectional | Community | Urban | India | Undergraduate | Not specified | 20.2 (4.6) | 63 |
| Stubbs et al., 2017. (ID-1620) | Cross-sectional | Community | Mixed | Bangladesh, India, Nepal, Pakistan, Sri Lanka | Mixed | Not specified | NA | 49.8 |
| Stubbs et al., 2016. (ID-1621) | Cross-sectional | Community | Mixed | Bangladesh, India, Nepal, Pakistan, Sri Lanka | Mixed | Not specified | NA | 49.9 |
| Susheela et al., 2018. (ID-1648) | Cross-sectional | Community | Rural | India | Mixed | Not specified | NA | 42.2 |
| Syed et al., 2018.  (ID-1652) | Cross-sectional | Community | Urban | Pakistan | Undergraduate | Not specified | 19.3 (1.19) | 24.7 |
| Tay et al., 2017.  (ID-1662) | Cross-sectional | Community | Mixed | Sri Lanka | Mixed | Not specified | 41.2 (0.21) | 27 |
| Thirthahalli et al., 2014. (ID-1678) | Cross-sectional | Community | Rural | India | Mixed | Not specified | NA | 30 |
| Tiwari et al., 2013. (ID-1690) | Cross-sectional | Community | Rural | India | NA | Lower middle income | 67.8 (5.9) | 47.4 |
| Tripathi et al., 2016. (ID-1704) | Cross-sectional | Primary care center | Mixed | India | NA | Not specified | NA | 33.7 |
| Wahlin et al., 2015. (ID-1753) | Cross-sectional | Community | Rural | Bangladesh | Mixed | Not specified | 69.57 (7.09) | 45 |
| Waqas et al., 2017. (ID-1761) | Cross-sectional | Tertiary care center | Mixed | Pakistan | Mixed | Not specified | NA | 47.2 |
| Wickramasinghe et al., 2016.  (ID-1772) | Cross-sectional | Community | Urban | Sri Lanka | NA | Not specified | 39.6 (9.2) | 83.4 |
| Wilkerson et al., 2018. (ID-1775) | Cross-sectional | Community | Urban | India | Mixed | Not specified | NA | 100 |
| Zalavadiya et al., 2017. (ID-1818) | Cross-sectional | Community | Urban | India | NA | Not specified | NA | NA |
| Zavos et al., 2015. (ID-1822) | Cross-sectional | Community | Urban | Sri Lanka | Mixed | Not specified | Twin=34,  Singleton=43 | Twins=48.2,  Non-twin=45.6 |
| Zubair et al., 2015. (ID-1828) | Cross-sectional | Other | Urban | Pakistan | High School | Not specified | NA | 100 |
| Axinn et al., 2013. (ID-MS 2) | Cross-sectional | Community | Rural | Nepal | Mixed | Not specified | Men=42, Women=34 | 41.3 |
| Ayub et al., 2009.  (ID-MS 3) | Cross-sectional | Primary care center | Urban | Pakistan | Mixed | Not specified | 34.41 (10.35) | 0 |
| Bhowmik et al., 2012. (ID-MS 4) | Cross-sectional | Community | Semi Urban | Bangladesh | NA | Not specified | Male=43.6(42.8-44.5), Female=40.2(39.8-40.07) | 36.7 |
| Fitch et al., 2017.  (ID-MS 5) | Cross-sectional | Community | Urban | Bangladesh | Mixed | Lower middle income | Garment workers=27.8(7.1),  Non-Garment Workers=32.7(8.9) | 0 |
| Goyal et al., 2011. (ID-MS 6) | Cross-sectional | Other | Mixed | India | Mixed | Not specified | 36.38 | 96 |
| Jayasuriya et al., 2016. (ID-MS 7) | Cross-sectional | Community | National | Sri Lanka | Mixed | Not specified | NA | 51 |
| Kane et al., 2017.  (ID-MS 8) | Cross-sectional | Community | Mixed | Nepal | Mixed | Not specified | 42.1 (5.8) | 41.5 |
| Kohrt et al., 2012. (ID-MS 9) | Longitudinal | Community | Mixed | Nepal | Mixed | Not specified | NA | Pre-Conflict=57.9,  Post-Conflict=56.4 |
| Kumar et al., 2013.  (ID-MS 10) | Cross-sectional | Community | Rural | India | Mixed | Not specified | 25.3 (9.0) | 52.3 |
| Reddy et al., 2012.  (ID-MS 12) | Cross-sectional | Community | Rural | India | Mixed | Not specified | NA | 50 |
| Siriwardhana et al., 2013.  (ID-MS 13) | Cross-sectional | Refugee setting | Rural | Sri Lanka | Mixed | Not specified | 37.1 (0.57) | 36.9 |
| Vankar et al., 2014.  (ID-MS 14) | Cross-sectional | Community | Urban | India | Undergraduate | Not specified | NA | 53.8 |
| Verma et al., 2010.  (ID-MS 15) | Cross-sectional | Community | Mixed | India | Mixed | Not specified | Female sex workers=30 (7.4), Male migrant workers= 26.5(5.5), Clients of Female sex workers= 26.5(5.1) | 69 |
| Saikia et al., 2016.  (ID-MS 16) | Cross-sectional | Community | Urban | India | NA | Not specified | NA | 46.5 |
| Sandhya et al., 2010.  (ID-MS 17) | Cross-sectional | Community | Rural | India | NA | Not specified | NA | 40.2 |
| Chauhan et al., 2016.  (ID-MS 18) | Cross-sectional | Community | Rural | India | NA | Not specified | NA | 33.8 |
| Mahi Rk et al., 2011.  (ID-MS 19) | Cross-sectional | Community | Rural | India | Mixed | Not specified | 39.02 (6.31) | 53.2 |
| Manjubhashini et al., 2013.  (ID-MS 20) | Cross-sectional | Community | Mixed | India | Mixed | Not specified | NA | 40 |
| Gautam et al., 2011.  (ID-MS 21) | Cross-sectional | Community | Mixed | Nepal | Mixed | Not specified | 69.9 (8.1) | 50.5 |
| Varshney et al., 2014.  (ID-MS 22) | Cross-sectional | Community | Mixed | India | Mixed | Not specified | NA | 40.2 |
| Ali et al., 2014.  (ID-MS 23) | Cross-sectional | Community | Urban | Pakistan | Undergraduate | Not specified | 20.4 (1.58) | 55.5 |
| Radhakrishnan et al., 2013.  (ID-MS 24) | Cross-sectional | Community | Rural | India | NA | Not specified | NA | 42 |
| Hakmaosa et al., 2015.  (ID-MS 26) | Cross-sectional | Community | Rural | India | Mixed | Not specified | NA | 40.3 |

CI= Confidence Interval, NA= Not available

***Table 1: Socio-demographic characteristics of the study populations.***

**References of Included Studies:**

1. Abbas, A., Rizvi, S.A., Hasan, R., Aqeel, N., Khan, M., Bhutto, A., Khan, Z. and Mannan, Z., 2015. The prevalence of depression and its perceptions among undergraduate pharmacy students. Pharmacy Education. 15, 57-63. doi: 10.4103/0253-7176.135372
2. Adhikari, A., Dutta, A., Sapkota, S., Chapagain, A., Aryal, A. and Pradhan, A., 2017. Prevalence of poor mental health among medical students in Nepal: a cross-sectional study. BMC Med Educ. 17, 232. doi: 10.1186/s12909-017-1083-0.
3. Ahmed, M.S., Walvekar, P.R., Chate, S.S. and Mallapur, M.D., 2016. Utility of Geriatric depression Scale-15 for assessment of depression among elderly: a cross sectional study. Indian Journal of Public Health Research & Development, 7, 150-154.
4. Akbar, S., Tiwari, S.C., Tripathi, R.K., Pandey, N.M. and Kumar, A., 2018. Prevalence of psychiatric illness among residents of old age homes in Northern India. J Neurosci Rural Pract. 9, 193-196. doi: 10.4103/jnrp.jnrp_340_17.
5. Akhtar, H., Khan, A.M., Vaidhyanathan, K.V., Chhabra, P. and Kannan, A.T., 2013. Socio-demographic predictors of depression among the elderly patients attending outpatient departments of a tertiary hospital in North India. Int J Prev Med. 4, 971–975.
6. Albers, H.M., Kinra, S., Krishna, K.R., Ben-Shlomo, Y. and Kuper, H., 2016. Prevalence and severity of depressive symptoms in relation to rural-to-urban migration in India: a cross-sectional study. BMC Psychol. 4, 47. doi:[10.1186/s40359-016-0152-1](https://doi.org/10.1186/s40359-016-0152-1).
7. Khan, S.A., Farooq, S. and Bano, A., 2012. Anxiety and depression in nurses working in government tertiary care teaching hospitals of Peshawar Khyber Pakhtunkhwa and their relationship with job stress. JPMI. 26, 34-38.
8. Ali, M., Farooq, N., Bhatti, M.A. and Kuroiwa, C., 2012. Assessment of prevalence and determinants of posttraumatic stress disorder in survivors of earthquake in Pakistan using Davidson Trauma Scale. J. jad. 136, 238-243. doi: 10.1016/j.jad.2011.12.023.
9. Altaf, A., Khan, M., Shah, S.R., Fatima, K., Tunio, S.A., Hussain, M., Khan, M.A., Shaikh, M.A. and Arshad, M.H., 2015. Sociodemographic pattern of depression in urban settlement of Karachi, Pakistan. JCDR. 9, doi: 10.7860/JCDR/2015/12611.6093.
10. Alvi, A.S. and Safdar, S., 2017. Depression and risk factors among elderly population of central Punjab, Pakistan. RMJ. 42, 571-574.
11. Alvi, T., Assad, F., Ramzan, M. and Khan, F.A., 2010. Depression, anxiety and their associated factors among medical students. JCPSP. 20, 122-6.
12. Archana, P.S., Das, S., Philip, S., Philip, R.R., Joseph, J., Punnoose, V.P. and Lalithambika, D.P., 2017. Prevalence of depression among middle aged women in the rural area of Kerala. J.ajp. 29, 154-159. doi: 10.1016/j.ajp.2017.05.016..
13. Atif, M., Bashir, A., Saleem, Q., Hussain, R. and Scahill, S., 2016. Health-related quality of life and depression among medical sales representatives in Pakistan. SpringerPlus, 5, 1048. doi: 10.1186/s40064-016-2716-1.
14. Avasthi, A., Basu, D., Subodh, B.N., Gupta, P.K., Malhotra, N., Rani, P. and Sharma, S., 2017. Substance use and dependence in the Union Territory of Chandigarh: Results of a household survey using a multistage stratified random sample. Indian J Psychiatry. 59. 275. doi: 10.4103/psychiatry.IndianJPsychiatry_326_16.
15. Avasthi, A., Basu, D., Subodh, B.N., Gupta, P.K., Sidhu, B.S., Gargi, P.D., Sharma, A., Ghosh, A. and Rani, P., 2018. Epidemiology of substance use and dependence in the state of Punjab, India: Results of a household survey on a statewide representative sample. j. ajp. 33,18-29. doi: 10.1016/j.ajp.2018.02.017.
16. Ball, H.A., Siribaddana, S.H., Kovas, Y., Glozier, N., McGuffin, P., Sumathipala, A. and Hotopf, M., 2010. Epidemiology and symptomatology of depression in Sri Lanka: a cross-sectional population-based survey in Colombo District. j. jad. 123, 188-196. doi: 10.1016/j.jad.2009.08.014.
17. Barua, A. and Kar, N., 2010. Screening for depression in elderly Indian population. Indian j psychiatry. 52, 150. doi: 10.4103/0019-5545.64595.
18. Basnet, S., Kandel, P. and Lamichhane, P., 2018. Depression and anxiety among war-widows of Nepal: a post-civil war cross-sectional study. Psychol Health Med. 23, 141-153. doi: 10.1080/13548506.2017.1338735.
19. Bawa, M.S. and Srivastav, M., 2013. Study the epidemiological profile of taxi drivers in the background of occupational environment, stress and personality characteristics. Indian J Occup Environ Med. 17, 108- 113.
20. Behera, P., Sharan, P., Mishra, A.K., Nongkynrih, B., Kant, S. and Gupta, S.K., 2016. Prevalence and determinants of depression among elderly persons in a rural community from northern India. Nmji. 29, 129- 135.
21. Bhamani, M.A., Karim, M.S. and Khan, M.M., 2013. Depression in the elderly in Karachi, Pakistan: a cross sectional study. BMC psychiatry, 13, 181.
22. Bhandari, P.M., Neupane, D., Rijal, S., Thapa, K., Mishra, S.R. and Poudyal, A.K., 2017. Sleep quality, internet addiction and depressive symptoms among undergraduate students in Nepal. BMC psychiatry, 17, 106. doi: 10.1186/s12888-017-1275-5.
23. Bhat, R.M. and Rangaiah, B., 2015. Exposure to armed conflict and prevalence of posttraumatic stress symptoms among young adults in Kashmir, India. Journal of Aggression, Maltreatment & Trauma. 24, 740-752. doi: 10.1080/10926771.2015.1062449.
24. Bishwajit, G., O’Leary, D.P., Ghosh, S., Yaya, S., Shangfeng, T. and Feng, Z., 2017. Physical inactivity and self-reported depression among middle-and older-aged population in South Asia: World health survey. BMC geriatrics. 17, 100. doi: 10.1186/s12877-017-0489-1.
25. Bishwajit, G., O’Leary, D.P., Ghosh, S., Sanni, Y., Shangfeng, T. and Zhanchun, F., 2017. Association between depression and fruit and vegetable consumption among adults in South Asia. BMC psychiatry. 17, 15. doi: 10.1186/s12888-017-1198-1.
26. Biswas, B., Haldar, A., Dasgupta, A., Sembiah, S., Karmakar, A. and Mallick, N., 2018. An Epidemiological Study on Burden of Psychological Morbidities and Their Determinants among Undergraduate Medical Students of a Government Medical College of Eastern India. Indian J Comm Health. 30, 3.
27. Boralingaiah, P., Bettappa, P. and Kashyap, S., 2012. Prevalence of psycho-social problems among elderly in urban population of Mysore city, Karnataka, India. Indian J Psychol Med. 34, 360. doi: 10.4103/0253-7176.108221.
28. Brinda, E.M., Rajkumar, A.P., Attermann, J., Gerdtham, U.G., Enemark, U. and Jacob, K.S., 2016. Health, social, and economic variables associated with depression among older people in low and middle income countries: world health organization study on global AGEing and adult health. Am J Geriatr Psychiatry. 24, 1196-1208. doi: 10.1016/j.jagp.2016.07.016.
29. Budhathoki, N., Shrestha, M.K., Acharya, N. and Manandhar, A., 2010. Subantance use among third year medical students of Nepal. [J Nepal Health Res Counc.](https://www.ncbi.nlm.nih.gov/pubmed/?term=Substance+Use+Among+Third+year+Medical+Students+of+Nepal) 8, 15-8.
30. Bukhari, S.R. and Khanam, S.J., 2015. Prevalence of depression in university students belonging to different socioeconomic status. JPMI. 29, 156-9.
31. Bushra, R. and Aslam, N., 2010. Prevalence of depression in Karachi, Pakistan. omj. 25, doi:10.5001/omj.2010.100.
32. Buvneshkumar, M., John, K.R. and Logaraj, M., 2018. A study on prevalence of depression and associated risk factors among elderly in a rural block of Tamil Nadu. ijph. 62, 89. doi: 10.4103/ijph.IJPH_33_17.
33. Chaudhuri, S.B., Mandal, P.K., Chakrabarty, M., Bandyopadhyay, G. and Bhattacherjee, S., 2017. A study on the prevalence of depression and its risk factors among adult population of Siliguri subdivision of Darjeeling district, West Bengal. jfmpc. 6, 351. doi: 10.4103/jfmpc.jfmpc_326_16.
34. Chellaiyan, V.G., Ali, F.L. and Maruthappapandian, J., 2018. Association between Sedentary Behaviour and Depression, Stress and Anxiety among Medical School Students in Chennai, India. JCDR. 12, doi: 10.7860/JCDR/2018/37129.12216.
35. Desai, N.G., Singh, V., Tripathi, C.B., Kumar, P., Jha, S., Chauhan, A., Samani, M., Tilwani, M.P., Gupta, D.K., Jahanara, M.G. and Sinha, U.K., 2017, January. Myth of PTSD-Long term epidemiological data on Psychiatric Disorders from Modified cohort study following a major natural disaster in India, Gujarat earthquake, 2001. indian j psychiatry. (Vol. 59, No. 6, S146-S146).
36. Deswal, B.S. and Pawar, A., 2012. An epidemiological study of mental disorders at Pune, Maharashtra. Indian J Community Med. 37, 116. doi: 10.4103/0970-0218.96097: 10.4103/0970-0218.96097.
37. Doherty, S., Hulland, E., Lopes-Cardozo, B., Kirupakaran, S., Surenthirakumaran, R., Cookson, S. and Siriwardhana, C., 2019. Prevalence of mental disorders and epidemiological associations in post-conflict primary care attendees: a cross-sectional study in the Northern Province of Sri Lanka. BMC psychiatry. 19, 83. doi: 10.1186/s12888-019-2064-0.
38. e Kalsoom, U. and Farid, K., 2014. Substance use among students of professional institutes of khyber pakhtunkhwa. JPMI. 28, 53-7.
39. Ehring, T., Razik, S. and Emmelkamp, P.M., 2011. Prevalence and predictors of posttraumatic stress disorder, anxiety, depression, and burnout in Pakistani earthquake recovery workers. j.psychres.185, 161-166. doi: 10.1016/j.psychres.2009.10.018.
40. Esie, P., Osypuk, T.L., Schuler, S.R. and Bates, L.M., 2019. Intimate partner violence and depression in rural Bangladesh: Accounting for violence severity in a high prevalence setting. J.ssmph. 7, 100368. doi: 10.1016/j.ssmph.2019.100368.
41. Firdaus, G., & Ahmad, A., 2014. Temporal variation in risk factors and prevalence rate of depression in urban population: does the urban environment play a significant role?. Int J Mental Health Prom. 16, 279-288. https://doi.org/10.1080/14623730.2014.931068
42. Fitch, T. J., Yu, X., Chien, L. C., Karim, M. M., & Alamgir, H., 2018. Traumatic life events and development of post-traumatic stress disorder among female factory workers in a developing country. Int J Soc Psych. 64, 351-358. https://doi.org/10.1177/0020764018761502.
43. Ghayas, S., Shamim, S., Anjum, F., & Hussain, M., 2014. Prevalence and severity of depression among undergraduate students in Karachi, Pakistan: A cross sectional study. Trop J Pharma Res. 13, 1733-1738. https://doi.org/10.4314/tjpr.v13i10.24
44. Ghimire, S., Baral, B. K., Pokhrel, B. R., Pokhrel, A., Acharya, A., Amatya, D., & Mishra, S. R., 2018. Depression, malnutrition, and health-related quality of life among Nepali older patients. BMC Ger. 18, 191. https://doi.org/10.1186/s12877-018-0881-5
45. Gitay, M. N., Fatima, S., Arshad, S., Arshad, B., Ehtesham, A., Baig, M. A., & Haque, Z., 2019. Gender Differences and Prevalence of Mental Health Problems in Students of Healthcare Units. Com Mental Health J. 55, 849-853. https://doi.org/10.1007/s10597-018-0304-2
46. Goldberg, D. P., Reed, G. M., Robles, R., Minhas, F., Razzaque, B., Fortes, S., & Dowell, A. C., 2017. Screening for anxiety, depression, and anxious depression in primary care: a field study for ICD-11 PHC. J Aff Dis, 213. 199-206. https://doi.org/10.1016/j.jad.2017.02.025
47. Goswami, S., Deshmukh, P. R., Pawar, R., Raut, A. V., Bhagat, M., & Mehendale, A. M., 2017. Magnitude of depression and its correlates among elderly population in a rural area of Maharashtra: A cross-sectional study. J Fam Med Prim Care.6, 803. https://doi.org/10.4103/jfmpc.jfmpc_97_17
48. Guerra, M., Prina, A. M., Ferri, C. P., Acosta, D., Gallardo, S., Huang, Y., & Salas, A., 2016. A comparative cross-cultural study of the prevalence of late life depression in low and middle income countries. J Aff Dis. 190, 362-368. https://doi.org/10.1016/j.jad.2015.09.004
49. Gupta, S., Sarpal, S. S., Kumar, D., Kaur, T., & Arora, S., 2013. Prevalence, pattern and familial effects of substance use among the male college students–a North Indian study. J Clinic Diagn Res. 7, 1632. https://doi.org/10.7860/JCDR/2013/6441.3215
50. Hashmi, S., Petraro, P., Rizzo, T., Nawaz, H., Choudhary, R., Tessier-Sherman, B.,& Nawaz, H., 2011. Symptoms of anxiety, depression, and posttraumatic stress among survivors of the 2005 Pakistani earthquake. Disas Med Pub Health Prep. 5, 293-299.  <https://doi.org/10.1001/dmp.2011.81>
51. Housen, T., Lenglet, A., Ariti, C. et al., 2017. Prevalence of anxiety, depression and post-traumatic stress disorder in the Kashmir Valley. BMJ Glob Health. 2, e000419. doi:10.1136/bmjgh-2017-000419
52. Husain, F., Anderson, M., Cardozo, B. et al., 2011. Prevalence of War-Related Mental Health Conditions and Association with Displacement Status in Postwar Jaffna District, Sri Lanka. Jama. 306, 522–531. doi: 10.1001/jama.2011.1052
53. Indu, P., Anilkumar, T., Pisharody, R., Russell, P., Raju, D., & Sarma, P. et al., 2017. Prevalence of depression and past suicide attempt in primary care. Asian J Psychiatr. 27, 48-52. doi: 10.1016/j.ajp.2017.02.008
54. Iqbal, S., Gupta, S., & Venkatarao, E., 2015. Stress, anxiety & depression among medical undergraduate students & their socio-demographic correlates. IIndian J Med Res. 141, 354-357. doi:10.4103/0971-5916.156571
55. Jadoon, N.A., Yaqoob, R., Raza, A., Shehzad, M.A., & Zeshan, S.C., 2010. Anxiety and depression among medical students: a cross-sectional study. J Pak Med Assoc. 60, 699-702
56. Jaisoorya, T.S., Reddy, Y.J., Nair, B.S., Rani, A., Menon, P.G., & Revamma, M. et al., 2017. Prevalence and correlates of obsessive-compulsive disorder and subthreshold obsessive-compulsive disorder among college students in Kerala, India. Indian J Psychiatry. 59, 56-62. doi: 10.4103/0019-5545.204438
57. MohsinJaved, S.B.A., Ullah, A., & Matee, S., 2015. Anxiety and depressive symptoms in primary caregivers of patients with severe depression: A snapshot from a military mental health care facility. Rawal Medical Journal. 40.
58. Jonas, J.B., Nangia, V., Rietschel, M., Paul, T., Behere, P., & Panda-Jonas, S., 2014. Prevalence of depression, suicidal ideation, alcohol intake and nicotine consumption in rural Central India. The Central India Eye and Medical Study. PLoS One. 9. doi: 10.1371/journal.pone.0113550
59. Kamal, S.M., Islam, M.A., & Raihan, M.A., 2011. Differentials of tobacco consumption and its effect on illicit drug use in rural men in Bangladesh. Asia Pac J Public Health. 23, 349-362. doi: 10.1177/1010539509345388
60. Kamble, S.V., Dhumale, G.B., Goyal, R.C., Phalke, D.B., & Ghodke, Y.D., 2009. Depression among elderly persons in a primary health centre area in Ahmednagar, Maharastra. Indian J Public Health. 53, 253-55.
61. Kar, S.K., Sharma, E., Agarwal, V., Singh, S.K., Dalal, P.K., & Singh, A. et al., 2018. Prevalence and pattern of mental illnesses in Uttar Pradesh, India: Findings from the National Mental Health Survey 2015–16. Asian J Psychiatr. 38, 45-52. doi: 10.1016/j.ajp.2018.10.023
62. Kato, T., 2016. Relationship between coping flexibility and the risk of depression in Indian adults. Asian J Psychiatr. 24, 130-134. doi:10.1016/j.ajp.2016.09.008
63. Kausar, N., Khan, S.D., & Akram, B., 2015. Major depression in Jalal Pur Jattan, district Gujrat, Pakistan: Prevalence and gender differences. J Pak Med Assoc. 65, 292-295.
64. Khaltar, A., Priyadarshani, N.G., Delpitiya, N.Y., Jayasinghe, C., Jayasinghe, A., Arai, A., & Tamashiro, H., 2017. Depression among older people in Sri Lanka: With special reference to ethnicity. Geriatr Gerontol Int. 17, 2414-2420. doi: 10.1111/ggi.13090
65. Khan, A.A., Haider, G., Sheikh, M.R., Ali, A. F., Khalid, Z., Tahir, M. et al., 2016. Prevalence of post-traumatic stress disorder due to community violence among university students in the world’s most dangerous megacity: A cross-sectional study from Pakistan. J Interpers Violence. 31, 2302-2315. doi: 10.1177/0886260515575605
66. Khan, M.S., Ahmed, U., Adnan, M., Khan, M.A., & Bawany, F.I., 2013. Frequency of generalised anxiety disorder and associated factors in an urban settlement of Karachi. J Pak Med Assoc. 63, 1451-1455.
67. Khanal, P., Ghimire, R.H., Gautam, B., Dhungana, S.K., Parajuli, P., Jaiswal, A.K., & Khanal, B., 2010. Substance use among medical students in Kathmandu valley. JNMA J Nepal Med Assoc. 50, 267-72. doi: 10.31729/jnma.26
68. Khanzada, F.J., Soomro, N., & Khan, S.Z., 2015. Association of physical exercise on anxiety and depression amongst adults. J Coll Physicians Surg Pak. 25, 546-548.
69. Kohli, C., Kishore, J., Agarwal, P., & Singh, S.V., 2013. Prevalence of unrecognised depression among outpatient department attendees of a rural hospital in Delhi, India.J Clin Diagn Res. 7, 1921-1925. doi: 10.7860/jcdr/2013/6449.3358
70. Kohrt, B.A., Speckman, R.A., Kunz, R.D., Baldwin, J.L., Upadhaya, N., Acharya, N.R. et al., 2009. Culture in psychiatric epidemiology: using ethnography and multiple mediator models to assess the relationship of caste with depression and anxiety in Nepal. Annals of human biology. 36, 261-280. doi: 10.1080/03014460902839194
71. Kulkarni, R.S., & Shinde, R.L., 2015. Depression and its associated factors in older Indians: a study based on Study of Global Aging and Adult Health (SAGE)–2007. J Aging Health. 27, 622-649. doi: 10.1177/0898264314556617
72. Kumar, G.S., Jain, A., & Hegde, S., 2012. Prevalence of depression and its associated factors using Beck Depression Inventory among students of a medical college in Karnataka. Indian J Psychiatry. 54, 223-226. doi: 10.4103/0019-5545.102412
73. Kumar, S., Mehrotra, D., Mishra, S., Goel, M.M., Kumar, S., Mathur, P., et al., 2015. Epidemiology of substance abuse in the population of Lucknow. J Oral Biol Craniofac Res. 5, 128-133. doi: 10.1016/j.jobcr.2015.08.010
74. Kumar, S.G., Kattimani, S., Sarkar, S., & Kar, S.S., 2017. Prevalence of depression and its relation to stress level among medical students in Puducherry, India. Ind Psychiatry J. 26, 86-90. doi:10.4103/ipj.ipj_45_15
75. Kunwar, D., Risal, A., & Koirala, S., 2016. Study of depression, anxiety and stress among the medical students in two medical colleges of Nepal. Kathmandu Univ Med J. 53, 22-6.
76. Lal, D., Sidhu, T.K., & Coonar, P.P.S., 2017. Prevalence and pattern of substance abuse among drivers in Punjab and Himachal Pradesh. Journal Of Evolution Of Medical And Dental Sciences. 6, 3686-3693. doi: 10.14260/jemds/2017/796
77. Lam, M.S., Fitzpatrick, A.L., Shrestha, A., Karmacharya, B.M., Koju, R., & Rao, D., 2017. Determining the Prevalence of and Risk Factors for Depressive Symptoms among Adults in Nepal. Int J Noncommun Dis. 2, 18-26. doi: 10.4103/jncd.jncd_34_16
78. Lee, C.H., Ko, A.M.S., Warnakulasuriya, S., Yin, B.L., Zain, R.B. et al., 2011. Intercountry prevalences and practices of betel‐quid use in south, southeast and eastern Asia regions and associated oral preneoplastic disorders: an international collaborative study by Asian betel‐quid consortium of south and east Asia. Int J Cancer. 129, 1741-1751. doi: 10.1002/ijc.25809
79. Luitel, N.P., Baron, E.C., Kohrt, B.A., Komproe, I.H., & Jordans, M.J., 2018. Prevalence and correlates of depression and alcohol use disorder among adults attending primary health care services in Nepal: a cross sectional study. BMC Health Serv Res. 18, 215. doi: 10.1186/s12913-018-3034-9
80. Luitel, N.P., Jordans, M.J., Sapkota, R.P., Tol, W.A., Kohrt, B.A., Thapa, S.B. et al. 2013. Conflict and mental health: a cross-sectional epidemiological study in Nepal. Social psychiatry and psychiatric epidemiology. 48, 183-193. doi: 10.1007/s00127-012-0539-0
81. Luni, F.K., Ansari, B., Jawad, A., Dawson, A., & Baig, S.M., 2009. Prevalence of depression and anxiety in a village in Sindh. J Ayub Med Coll Abbottabad. 21, 68-72.
82. Malhotra, R., Chan, A., & Østbye, T., 2010. Prevalence and correlates of clinically significant depressive symptoms among elderly people in Sri Lanka: findings from a national survey. Int Psychogeriatr. 22, 227-236. doi: 10.1017/s1041610209990871
83. Malik, M.K., Jacob, K.S., 2015. Psychological morbidity among co-residents of older people in rural South India: Prevalence and risk factors. Int J Soc Psychiatry. 61, 183-187. doi: 10.1177/0020764014539287
84. Mathias, K., Goicolea, I., Kermode, M., Singh, L., Shidhaye, R., & San Sebastian, M., 2015. Cross-sectional study of depression and help-seeking in Uttarakhand, North India. BMJ open. 5, e008992. doi: 10.1136/bmjopen-2015-008992
85. Medhi, G.K., Mahanta, J., Kermode, M., Paranjape, R.S., Adhikary, R., Phukan, S.K., & Ngully, P., 2012. Factors associated with history of drug use among female sex workers (FSW) in a high HIV prevalence state of India. BMC public health. 12, 273. doi: 10.1186/1471-2458-12-273
86. Mubeen, S.M., Henry, D., & Qureshi, S.N., 2012. Prevalence of depression among community dwelling elderly in Karachi, Pakistan. Iranian journal of psychiatry and behavioral sciences. 6, 84.
87. Swarnalatha, N., 2013. The prevalence of depression among the rural elderly in Chittoor District, Andhra Pradesh. J Clin Diagn Res. 7, 1356-1360. doi: 10.7860/jcdr/2013/5956.3141
88. Nabi, N., Yousuf, A., & Iqbal, A., 2012. Prevalence of anxiety and depression among doctors working in a Private Hospital in Pakistan. ASEAN Journal of Psychiatry. 13, 13-19
89. Naeem, F., Ayub, M., Masood, K., Gul, H., Khalid, M., & Farrukh, A. et al., 2011. Prevalence and psychosocial risk factors of PTSD: 18months after Kashmir earthquake in Pakistan. J Affect Disord. 130, 268-274. doi: 10.1016/j.jad.2010.10.035
90. Nagoor, K., Darivemula, S.B., Reddy, N.B., Patan, S.K., Deepthi, C.S., & Chittooru, C. S., 2018. Prevalence of mental illness and their association with sociodemographic factors in the rural geriatric population in Chittoor, Andhra Pradesh, India: A community-based study. J Educ Health Promot. 7,165. doi: 10.4103/jehp.jehp_193_18
91. Nair, S.S., Raghunath, P., & Nair, S.S., 2015. Prevalence of psychiatric disorders among the rural geriatric population: a pilot study in Karnataka, India. Cent Asian J Glob Health. 4. doi: 10.5195/cajgh.2015.138
92. Natasha, K., Hussain, A., Azad Khan, A.K., & Bhowmik, B., 2015. Prevalence of depression and glucose abnormality in an urbanizing rural population of Bangladesh. Diabetes Metab J. 39, 218-229. doi: 10.4093/dmj.2015.39.3.218
93. Padda, P., Gupta, S., Singh, G., Singh, L., & Chawla, N., 2016. An epidemiological study of depression among college students in district Faridkot, Punjab, India. Indian Journal of Community Health. 28, 151-156.
94. Panthee, B., Panthee, S., Gyawali, S., & Kawakami, N., 2017. Prevalence and correlates of substance use among health care students in Nepal: a cross sectional study. BMC public health. 17, 950. doi: 10.1186/s12889-017-4980-6
95. Patel, P.A., Patel, P.P., Khadilkar, A.V., Chiplonkar, S.A., & Patel, A.D., 2017. Impact of occupation on stress and anxiety among Indian women. Women & health. 57, 392-401. doi: 10.1080/03630242.2016.1164273
96. Patel, S.K., Ganju, D., Prabhakar, P., & Adhikary, R., 2016. Relationship between mobility, violence and major depression among female sex workers: a cross-sectional study in southern India. BMJ open. 6, e011439. doi: 10.1136/bmjopen-2016-011439
97. Patel, S.K., Saggurti, N., Pachauri, S., & Prabhakar, P., 2015. Correlates of mental depression among female sex workers in Southern India. Asia Pac J Public Health. 27, 809-819. doi: 10.1177/1010539515601480
98. Perveen, S., Kazmi, S.F., & Rehman, A.U., 2016. Relationship between negative cognitive style and depression among medical students. J Ayub Med Coll Abbottabad. 28, 94-98.
99. Chawla, S., Gour, N., Goel, P.K., & Rohilla, R., 2018. Depression and its correlates among geriatric people: A community based study from Southern Haryana, India. Indian Journal of Community and Family Medicine. 4, 49-54. doi: 10.4103/2395-2113.251439
100. Poongothai, S., Pradeepa, R., Ganesan, A., & Mohan, V., 2009. Prevalence of depression in a large urban South Indian population—The Chennai Urban Rural Epidemiology study (CURES–70). PloS one. 4. doi: 10.1371/journal.pone.0007185
101. Prina, A.M., Ferri, C.P., Guerra, M., Brayne, C. and Prince, M., 2011. Co-occurrence of anxiety and depression amongst older adults in low-and middle-income countries: findings from the 10/66 study. Psychological Medicine. 41, 2047–2056. doi:10.1017/S0033291711000444.
102. Rajapakshe, O.B., Sivayogan, S. and Kulatunga, P.M., 2019. Prevalence and correlates of depression among older urban community‐dwelling adults in Sri Lanka. Psychogeriatrics. 19, 202-211. <https://doi.org/10.1111/psyg.12389>
103. Rajkumar, A.P., Thangadurai, P., Senthilkumar, P., Gayathri, K., Prince, M. and Jacob, K.S., 2009. Nature, prevalence and factors associated with depression among the elderly in a rural south Indian community. Int Psychogeriatr. 21, 372-378. <https://doi.org/10.1017/S1041610209008527>.
104. Rao, T.S., Darshan, M.S., Tandon, A., Raman, R., Karthik, K.N., Saraswathi, N., Das, K., Harsha, G.T., Krishna, V.S.T. and Ashok, N.C., 2014. Suttur study: An epidemiological study of psychiatric disorders in South Indian rural population. Indian J Psychiatry. 56, 238. doi: 10.4103/0019-5545.140618.
105. Rathod, M.S., Dixit, J.V., Goel, A.D. and Yadav, V., 2019. Prevalence of depression in an urban geriatric population in Marathwada region of Western India. Indian J Psychol Med. 41, 32-37. DOI: 10.4103/IJPSYM.IJPSYM_234_18.
106. Rathod, S.D., Luitel, N.P. and Jordans, M.J.D., 2018. Prevalence and correlates of alcohol use in a central Nepal district: secondary analysis of a population-based cross-sectional study. Glob Ment Health (Camb). 5, 1-11. doi:10.1017/gmh.2018.28.
107. Rathod, S.D., Nadkarni, A., Bhana, A. and Shidhaye, R., 2015. Epidemiological features of alcohol use in rural India: a population-based cross-sectional study. BMJ Open. 5, 1-8. <http://dx.doi.org/10.1136/bmjopen-2015-009802>.
108. Rathod, S.D., Roberts, T., Medhin, G., Murhar, V., Samudre, S., Luitel, N.P., Selohilwe, O., Ssebunnya, J., Jordans, M.J., Bhana, A. and Petersen, I., 2018. Detection and treatment initiation for depression and alcohol use disorders: facility-based cross-sectional studies in five low-income and middle-income country districts. BMJ Open. 8, 1-20. <http://dx.doi.org/10.1136/bmjopen-2018-023421>.
109. Risal, A., Manandhar, K., Linde, M., Steiner, T.J. and Holen, A., 2016. Anxiety and depression in Nepal: prevalence, comorbidity and associations. BMC Psychiatry. 16, 102. <https://doi.org/10.1186/s12888-016-0810-0>.
110. Saddichha, S. and Khess, C.R.J., 2010. Prevalence of tobacco use among young adult males in India: a community-based epidemiological study. Am J Drug Alcohol Abuse. 36, 73-77. <https://doi.org/10.3109/00952990903575814>.
111. Saeed, H., Saleem, Z., Ashraf, M., Razzaq, N., Akhtar, K., Maryam, A., Abbas, N., Akhtar, A., Fatima, N., Khan, K. and Rasool, F., 2018. Determinants of anxiety and depression among university students of Lahore. Int J Ment Health Addiction. 16, 1283-1298. https://doi.org/10.1007/s11469-017-9859-3.
112. Sagar, R., Pattanayak, R.D., Chandrasekaran, R., Chaudhury, P.K., Deswal, B.S., Singh, R.L., Malhotra, S., Nizamie, S.H., Panchal, B.N., Sudhakar, T.P. and Trivedi, J.K., 2017. Twelve-month prevalence and treatment gap for common mental disorders: Findings from a large-scale epidemiological survey in India. Indian J Psychiatry. 59, 46. doi: 10.4103/psychiatry.IndianJPsychiatry_333_16.
113. Sahoo, S. and Khess, C.R., 2010. Prevalence of depression, anxiety, and stress among young male adults in India: a dimensional and categorical diagnoses-based study. The Journal of nervous and mental disease. 198, 901-904. doi: 10.1097/NMD.0b013e3181fe75dc.
114. Salve, H., Goswami, K., Nongkynrih, B., Sagar, R. and Sreenivas, V., 2012. Prevalence of psychiatric morbidity at Mobile Health Clinic in an urban community in North India. Gen Hosp Psychiatry. 34, 121-126. doi:10.1016/j.genhosppsych.2011.09.009.
115. Senarath, U., Wickramage, K. and Peiris, S.L., 2014. Prevalence of depression and its associated factors among patients attending primary care settings in the post-conflict Northern Province in Sri Lanka: a cross-sectional study. BMC psychiatry. 14, 85.  doi: 10.1186/1471-244X-14-85.
116. Sengupta, P. and Benjamin, A.I., 2015. Prevalence of depression and associated risk factors among the elderly in urban and rural field practice areas of a tertiary care institution in Ludhiana. İndian journal of public health, 59, 3. Indian J Public Health. doi: 10.4103/0019-557X.152845.
117. Shidhaye, R., Gangale, S. and Patel, V., 2016. Prevalence and treatment coverage for depression: a population-based survey in Vidarbha, India. Soc Psychiatry Psychiatr Epidemiol. 51, 993-1003. doi: 10.1007/s00127-016-1220-9.
118. Shidhaye, R., Lyngdoh, T., Murhar, V., Samudre, S. and Krafft, T., 2017. Predictors, help-seeking behaviour and treatment coverage for depression in adults in Sehore district, India. BJPsych open. 3, 212-222. doi: 10.1192/bjpo.bp.116.004648.
119. Parvin, K., Al Mamun, M., Gibbs, A., Jewkes, R. and Naved, R.T., 2018. The pathways between female garment workers’ experience of violence and development of depressive symptoms. PloS one. 13, 1-17. doi: 10.1371/journal.pone.0207485.
120. Shrestha, G., Yadav, D.K., Sapkota, N., Baral, D., Yadav, B.K., Chakravartty, A. and Pokharel, P.K., 2017. Depression among inmates in a regional prison of eastern Nepal: a cross-sectional study. BMC psychiatry. 17, 348. doi: 10.1186/s12888-017-1514-9.
121. Simkhada, R., Wasti, S.P., Gc, V.S. and Lee, A.C., 2018. Prevalence of depressive symptoms and its associated factors in older adults: a cross-sectional study in Kathmandu, Nepal. Aging Ment Health. 22, 802-807. doi: 10.1080/13607863.2017.1310803.
122. Singh, A. and Lal, A., 2010. Prevalence of Depression Among Medical Students of a Private Medical College in India. J Health Allied Scs. 9, 8.
123. Stubbs, B., Koyanagi, A., Hallgren, M., Firth, J., Richards, J., Schuch, F., Rosenbaum, S., Mugisha, J., Veronese, N., Lahti, J. and Vancampfort, D., 2017. Physical activity and anxiety: a perspective from the World Health Survey. Journal of Affective Disorders. J Affect Disord. 208, 545-552. doi: 10.1016/j.jad.2016.10.028.
124. Stubbs, B., Koyanagi, A., Schuch, F.B., Firth, J., Rosenbaum, S., Veronese, N., Solmi, M., Mugisha, J. and Vancampfort, D., 2016. Physical activity and depression: a large cross‐sectional, population‐based study across 36 low‐and middle‐income countries. Acta Psychiatr Scand. 134, 546-556. doi: 10.1111/acps.12654.
125. Susheela, P. and Valsaraj, B.P., 2018. Depression, Perceived Loneliness and Partial Functional Impairment among Older Adults. Journal of Clinical & Diagnostic Research. 12, 1-5. DOI: 10.7860/JCDR/2018/34086.12096.
126. Syed, A., Ali, S.S. and Khan, M., 2018. Frequency of depression, anxiety and stress among the undergraduate physiotherapy students. Pak J Med Sci. 34, 468. doi: 10.12669/pjms.342.12298.
127. Tay, A.K., Jayasuriya, R., Jayasuriya, D. and Silove, D., 2017. Twelve-month trajectories of depressive and anxiety symptoms and associations with traumatic exposure and ongoing adversities: a latent trajectory analysis of a community cohort exposed to severe conflict in Sri Lanka. Transl psychiatry. 7, 1200.  doi: 10.1038/tp.2017.166.
128. Thirthahalli, C., Suryanarayana, S.P., Sukumar, G.M., Bharath, S., Rao, G.N. and Murthy, N.S., 2014. Proportion and factors associated with depressive symptoms among elderly in an urban slum in Bangalore. J Urban Health. 91, 1065-1075. doi: 10.1007/s11524-014-9903-6.
129. Tiwari, S.C., Srivastava, G., Tripathi, R.K., Pandey, N.M., Agarwal, G.G., Pandey, S. and Tiwari, S., 2013. Prevalence of psychiatric morbidity amongst the community dwelling rural older adults in northern India. Indian J Med Res. 138, 504.
130. Tripathi, A., Kallivayalil, R.A., Bhagabati, D. and Sorel, E., 2016. An exploratory multi-centric depression screening study in primary care setting from India. International Medical Journal. 23, 122-124.
131. Wahlin, Å., Palmer, K., Sternäng, O., Hamadani, J.D. and Kabir, Z.N., 2015. Prevalence of depressive symptoms and suicidal thoughts among elderly persons in rural Bangladesh. Int Psychogeriatr. 27, 1999-2008. doi: 10.1017/S104161021500109X.
132. Waqas, A., Raza, N., Zahid, T., Rehman, A., Hamid, T., Hanif, A., Jamal, M., Farrukh, A., Azam, A., Turk, M. and Chaudhry, M.A., 2018. Predictors of post-traumatic stress disorder among burn patients in Pakistan: The role of reconstructive surgery in post-burn psychosocial adjustment. Burns. 44, 620-625. doi: 10.1016/j.burns.2017.09.012.
133. Wickramasinghe, N.D., Wijesinghe, P.R., Dharmaratne, S.D. and Agampodi, S.B., 2016. The prevalence and associated factors of depression in policing: a cross sectional study in Sri Lanka. SpringerPlus. 5,1776. doi: 10.1186/s40064-016-3474-9
134. Wilkerson, J.M., Di Paola, A., Rawat, S., Patankar, P., Rosser, B.S. and Ekstrand, M.L., 2018. Substance use, mental health, HIV testing, and sexual risk behavior among men who have sex with men in the state of Maharashtra, India. AIDS Educ Prev. 30, 96-107. doi: 10.1521/aeap.2018.30.2.96
135. Zalavadiya, D.D., Banerjee, A., Sheth, A.M., Rangoonwala, M., Mitra, A. and Kadri, A.M., 2017. A comparative study of depression and associated risk factors among elderly inmates of old age homes and community of Rajkot: A Gujarati version of the geriatric depression scale-short form (GDS-G). Indian J Community Med. 42, 204-208. doi:10.4103/ijcm.IJCM_181_16
136. 1822. Zavos, H.M., Siribaddana, S., Ball, H.A., Lynskey, M.T., Sumathipala, A., Rijsdijk, F.V. and Hotopf, M., 2015. The prevalence and correlates of alcohol use and alcohol use disorders: a population based study in Colombo, Sri Lanka. BMC psychiatry. 15, 158. doi: 10.1186/s12888-015-0549-z
137. Zubair, U.B., Mansoor, S. and Rana, M.H., 2015. Prevalence of depressive symptoms and associated socio-demographic factors among recruits during military training. J R Army Med Corps. 161, 127-131. doi: 10.1136/jramc-2014-000253
138. Axinn, W.G., Ghimire, D.J., Williams, N.E. and Scott, K.M., 2013. Gender, traumatic events, and mental health disorders in a rural Asian setting. J Health Soc Behav. 54, 444-461. doi: 10.1177/0022146513501518
139. Ayub, M., Irfan, M., Nasr, T., Lutufullah, M., Kingdon, D. and Naeem, F., 2009. Psychiatric morbidity and domestic violence: a survey of married women in Lahore. Soc Psychiat Epidemiol. 44, 953. doi: 10.1007/s00127-009-0016-6
140. Bhowmik, B., Binte Munir, S., Ara Hossain, I., Siddiquee, T., Diep, L.M., Mahmood, S., Mahtab, H., Khan, A.K. and Hussain, A., 2012. Prevalence of type 2 diabetes and impaired glucose regulation with associated cardiometabolic risk factors and depression in an urbanizing rural community in bangladesh: a population-based cross-sectional study. Diabetes Metab J. 36, 422-432. doi: 10.4093/dmj.2012.36.6.422
141. Fitch, T.J., Moran, J., Villanueva, G., Sagiraju, H.K.R., Quadir, M.M. and Alamgir, H., 2017. Prevalence and risk factors of depression among garment workers in Bangladesh. Int J Soc Psychiatry. 63, 244-254. doi: 10.1177/0020764017695576
142. Goyal, S.K., Singh, P., Gargi, P.D., Goyal, S. and Garg, A., 2011. Psychiatric morbidity in prisoners. Indian J psychiatry. 53, 253. doi:10.4103/0019-5545.86819
143. Jayasuriya, D., Jayasuriya, R., Tay, A.K. and Silove, D., 2016. Associations of mental distress with residency in conflict zones, ethnic minority status, and potentially modifiable social factors following conflict in Sri Lanka: a nationwide cross-sectional study. Lancet Psychiatry. 3, 145-153. doi: 10.1016/S2215-0366(15)00437-X.
144. Kane, J.C., Luitel, N.P., Jordans, M.J.D., Kohrt, B.A., Weissbecker, I. and Tol, W.A., 2018. Mental health and psychosocial problems in the aftermath of the Nepal earthquakes: findings from a representative cluster sample survey. Epidemiol Psychiatr Sci. 27, 301-310. doi:10.1017/S2045796016001104
145. Kohrt, B.A., Hruschka, D.J., Worthman, C.M., Kunz, R.D., Baldwin, J.L., Upadhaya, N., Acharya, N.R., Koirala, S., Thapa, S.B., Tol, W.A. and Jordans, M.J., 2012. Political violence and mental health in Nepal: prospective study. Br J Psychiatry. 201, 268-275. doi:10.1192/bjp.bp.111.096222
146. Ganesh Kumar, S., Premarajan, K.C., Subitha, L., Suguna, E. and Vinayagamoorthy, V.K., 2013. Prevalence and pattern of alcohol consumption using alcohol use disorders identification test (AUDIT) in rural Tamil Nadu, India. J Clin Diagn Res. 7, 1637. doi:10.7860/JCDR/2013/5521.3216
147. Reddy, N.B., Pallavi, M., Reddy, N.N., Reddy, C.S., Singh, R.K. and Pirabu, R.A., 2012. Psychological morbidity status among the rural geriatric population of Tamil Nadu, India: A cross-sectional study. Indian J Psychol Med. 34, 227. doi:10.4103/0253-7176.106016
148. Siriwardhana, C., Adikari, A., Pannala, G., Siribaddana, S., Abas, M., Sumathipala, A. and Stewart, R., 2013. Prolonged internal displacement and common mental disorders in Sri Lanka: the COMRAID study. PloS one. 8. doi:10.1371/journal.pone.0064742
149. MS 14. Vankar, J.R., Prabhakaran, A. and Sharma, H., 2014. Depression and stigma in medical students at a private medical college. Indian J Psychol Med. 36, 246. doi:10.4103/0253-7176.135372
150. Verma, R.K., Saggurti, N., Singh, A.K. and Swain, S.N., 2010. Alcohol and sexual risk behavior among migrant female sex workers and male workers in districts with high in-migration from four high HIV prevalence states in India. AIDS Behav. 14, 31-39. doi:10.1007/s10461-010-9731-y
151. Saikia, A.M., Mahanta, N., Saikia, A.M., Deka, H., Boruah, B. and Mehanta, R., 2016. Depression in elderly: a community–based study from Assam. Indian J Basic Appl Med Res. 5, 42-48.
152. Sandhya, G.I., 2010. Geriatric depression and related factors-a cross sectional study from a rural community in South Kerala. J Ind Acad Geriatr. 6, 61-3.
153. Chauhan, P., Kokiwar, P.R., Shridevi, K. and Katkuri, S., 2016. A study on prevalence and correlates of depression among elderly population of rural South India.Int J Community Med Public Health. 3, 236-239. doi: 10.18203/2394-6040.ijcmph20151569
154. Rk, Mahi & Sharma, Arvind., 2011. An Epidemiological Survey of Alcohol and Drug Dependence in a Village of district Sangrur, Punjab. Delhi psychiatry journal. 14.
155. Manjubhashini, S., Krishnababu, G. and Krishnaveni, A., 2013. Epidemiological study of depression among population above 60 years in Visakhapatnam, India. International Journal of Medical Science and Public Health. 2, 695-703. doi: 10.5455/ijmsph.2013.030520133
156. Gautam, R. and Houde, S., 2011. Geriatric depression scale for community-dwelling older adults in Nepal. Asian j gerontol Geriatr. 6, 93-9.
157. Varshney, D.S., Semwal, J., Srivastava, A.K., Vyas, S. and Sati, H., 2014. Practices and socio cultural aspects of substance use among residents of a newly formed state: A cross sectional study in Dehradun. Natl J Med Res, 4, 330-6.
158. Ali, A., Rao, M.H., Ali, S., Ahmed, T., Safi, M., Malik, A. and Husan, B., 2014. Prevalence of anxiety and depression and their associated risk factors among engineering students in Karachi, Pakistan. International Journal of Emerging Technology and Advanced Engineering, 4, 52-55.
159. Radhakrishnan, S. and Nayeem, A., 2013. Prevalence of depression among geriatric population in a rural area in Tamilnadu. International journal of nutrition, pharmacology, neurological diseases, 3, 309.
160. Hakmaosa, A., Baruah, K.K., Baruah, R. and Hajong, S., 2015. Prevalence of depression among elderly in Rani block, Kamrup (rural) district, Assam. Indian J Appl Res, 5, 369-71.
